# Fluorescent small organic probes for biosensing

**DOI:** 10.1039/d0sc06928k

**Published:** 2021-01-18

**Authors:** Xue Tian, Lloyd C. Murfin, Luling Wu, Simon E. Lewis, Tony D. James

**Affiliations:** Department of Chemistry, University of Bath Bath BA2 7AY UK lw960@bath.ac.uk s.e.lewis@bath.ac.uk t.d.james@bath.ac.uk; School of Chemistry and Chemical Engineering, Henan Normal University Xinxiang 453007 P. R. China

## Abstract

Small-molecule based fluorescent probes are increasingly important for the detection and imaging of biological signaling molecules due to their simplicity, high selectivity and sensitivity, whilst being non-invasive, and suitable for real-time analysis of living systems. With this perspective we highlight sensing mechanisms including Förster resonance energy transfer (FRET), intramolecular charge transfer (ICT), photoinduced electron transfer (PeT), excited state intramolecular proton transfer (ESIPT), aggregation induced emission (AIE) and multiple modality fluorescence approaches including dual/triple sensing mechanisms (DSM or TSM). Throughout the perspective we highlight the remaining challenges and suggest potential directions for development towards improved small-molecule fluorescent probes suitable for biosensing.

## Abstract 2

Small-molecule based fluorescent probes are increasingly important for the detection and imaging of biological signaling molecules due to their simplicity, high selectivity and sensitivity, whilst being non-invasive, and suitable for real-time analysis of living systems.

## Graphical abstract

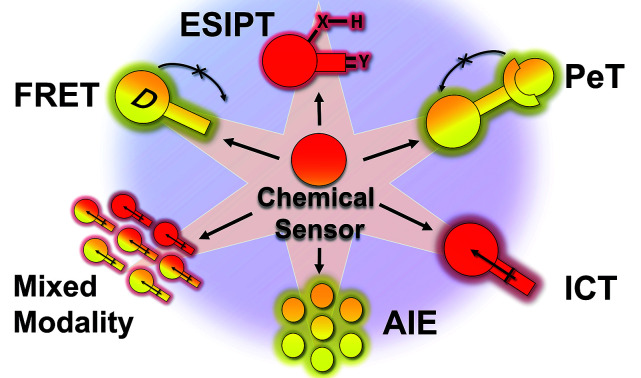

## 1. Introduction

**Scheme 1:**
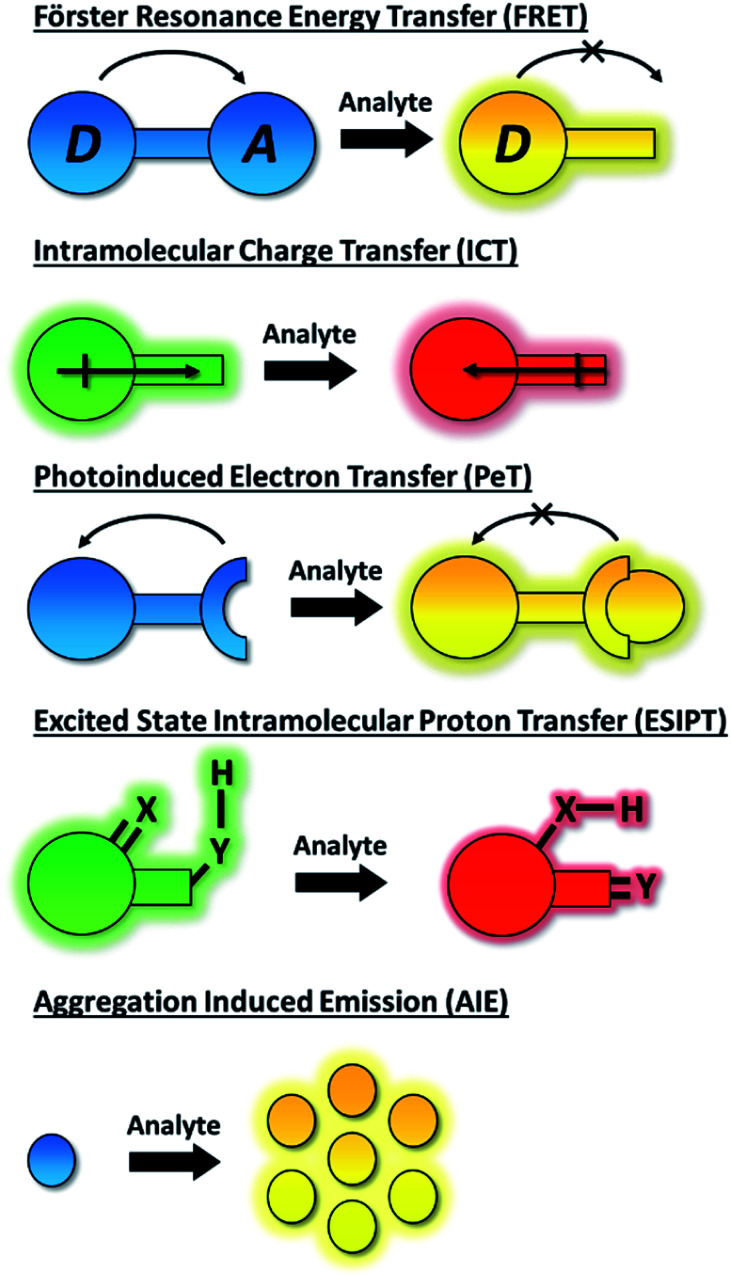
Schematic representation of the fluorescent mechanisms discussed in this perspective.

The dynamic chemical diversity of the numerous elements, ions and molecules that constitute the basis of life provides wide challenges and opportunities for research. Due to their high levels of sensitivity, fast response time, and technical simplicity, small molecule based fluorescent probes have been widely developed and applied to the detection of many biologically important analytes.^1–10^ In particular, fluorescent probes for biologically and/or environmentally important cations, anions, small neutral molecules, and biological macromolecules (such as protein and DNA) have been developed to complement the rapid development of microscopic imaging technologies. As such, fluorescent probes have been extensively used in diverse fields such as biology, physiology, medicine, pharmacology, and environmental sciences. Such probes exhibit changes in fluorescence intensities or emission wavelengths through one or more sensing mechanisms, including Förster resonance energy transfer (FRET),^11^ intramolecular charge transfer (ICT),^1^ photoinduced electron transfer (PeT),^12^ excited state intramolecular proton transfer (ESIPT),^13^ aggregation induced emission (AIE),^14,15^ or involve dual/triple sensing mechanisms (DSM or TSM) (Scheme 1).^16^ Importantly, the number of fluorescence based systems continues to expand exponentially, in order to keep pace with the ever expanding fluorescence imaging modalities, including super-resolution-based technologies such as Stochastic Optical Reconstruction Microscopy (STORM),^17^ PALM (photoactivated localization microscopy)^18^ and super-resolution photoacoustic imaging (PAI).^19–21^

An ideal fluorescence sensing system provides a reliable output response under analytical conditions which relates directly to the concentration of the analyte present. Such interactions can be reversible (the probe being described as a “chemosensor”) providing an accurate reflection of analyte concentration that may change over time. If the interaction process is irreversible (the probe being given the term “chemodosimeter”) the response will indicate only the maximum concentration of analyte present. Importantly, for fluorescent systems, the nature of the probe's response can also vary. In particular a turn-on fluorescence response upon analyte detection is desired, to differentiate the response from environmental quenching effects. Likewise, the use of turn on/off fluorescent probes that exhibit only a single emission maximum for quantifying target analytes may be fraught with difficulties due to interference from various analytically unrelated factors. Instruments' parameters, the microenvironment around the probe molecule, *etc.* can all interfere with the analysis.^22^ Significantly, by taking the advantage of the ratiometric approach these problems can be successfully overcome. The ratiometric fluorescence response depends on the intensity change of two or more emission bands of a probe species before and after the analyte recognition event. As such, it provides an effective internal reference that significantly improves sensitivity and quantification.

Amongst organic fluorescent probes, near-infrared (NIR, 650–900 nm) probes have proven to be especially advantageous when used *in vivo* or *in vitro*.^23–26^ Such probes allow for deep-tissue penetration, reduce interference from haemoglobin and water,^27^ and lessen the risk of photobleaching due to the lower energy of excitation required.^28^ Furthermore, autofluorescence of endogenous species is reduced since such species usually absorb or emit below 600 nm.^29^ The use of two-photon (TP) excitation, first described by Webb *et al.* in 1990,^30^ has been widespread in the world of NIR fluorescent probes.^31,32^ TP excitation occurs when two (typically NIR) photons of similar energy are absorbed by a molecule, and the combined energies promote it to an excited state suitable for fluorescence emission.^33^ TP fluorescence has the same advantages as NIR fluorescence, but in addition can generate fluorescence emissions in the visible region. Furthermore, it allows for specific localisation of excitation, since the absorption of two photons only happens within the focal plane, therefore potentially reducing photodamage.^34^ However, some evidence suggests that use of TPM for biological imaging can in fact lead to greater photobleaching (in comparison to single photon experiments), dependent on the power level at which the imaging is performed.^35^ However, the extent of photobleaching can be minimised by use of higher pulse repetition rates in preference to a continuous laser source.^36^

In this perspective, we will highlight the features of fluorescent probes that use a variety of different fluorescence mechanisms, with a focus on those that have shown promise in the realms of biological sensing and imaging. Moreover, we will highlight how fluorescent probes with optimized optical properties are powerful tools for biological research. For example, NIR emission and TP probes are useful to image target analytes in deep tissues. After detailing prominent examples using each fluorescence technique, a consideration of the advantages and disadvantages of that mode of fluorescence is also presented.

## 2. Förster resonance energy transfer (FRET) probes

**Fig. 1:**
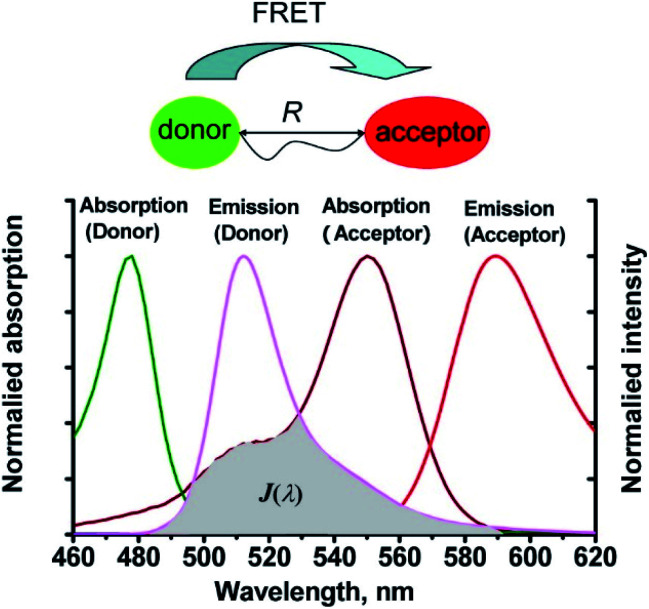
The mechanism of Förster resonance energy transfer (FRET). *R* is the distance between the energy donor and acceptor, *J*(*λ*) represents the degree of spectroscopic overlap between the donor emission and the acceptor absorption. Reproduced with permission from ref. 37. Copyright 2013, American Chemical Society.

Förster resonance energy transfer (FRET) is a non-radiative process in which excited dye donors transfer energy to ground state dye acceptors through a long range dipole–dipole interaction (Fig. 1).^37^ The donor moiety absorbs higher energy, shorter wavelength light, whereas the acceptor absorbs and emits lower energy, longer wavelength light. Considerations for designing FRET-based probes include: (1) the donor–acceptor pair should be within 10–100 Å of each other, (2) emission spectrum of the donor and the absorption spectrum of the acceptor should overlap, (3) donor emission moment, acceptor absorption moment, and the separation vector should be favourably oriented. At this point readers are directed to a recent review by James *et al.* covering FRET-based small-molecule fluorescent probes for the detection or imaging of cations, anions, small neutral molecules, biomacromolecules, cellular microenvironments and dual/multi-analyte responsive systems.^11^

**Scheme 2:**
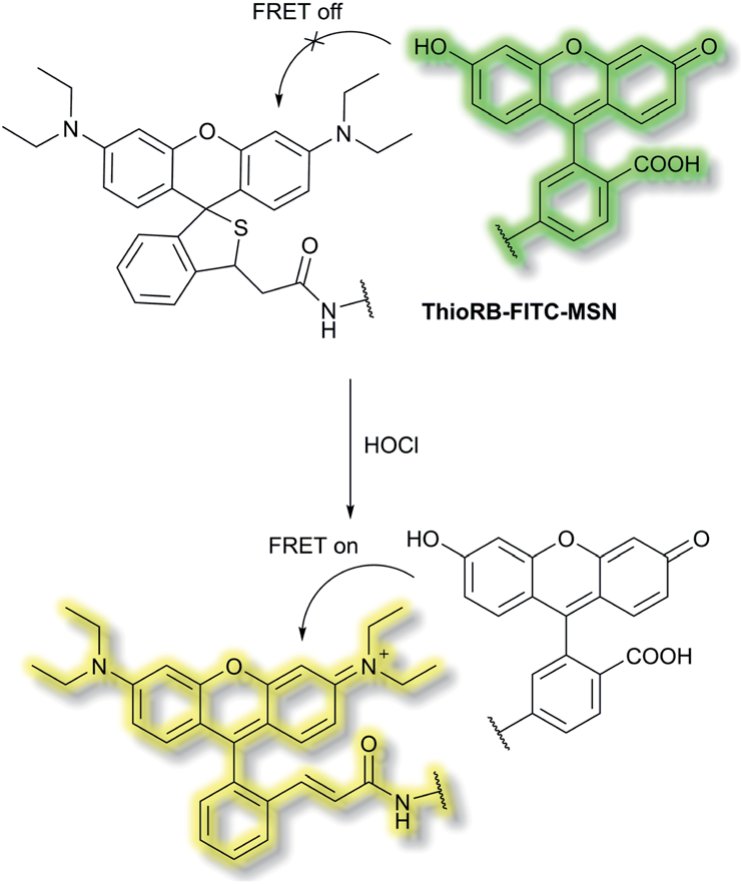
FRET-based ratiometric fluorescent probe **ThioRB-FITC-MSN** for the detection of HOCl.

Han *et al.* have developed a ratiometric probe **ThioRB-FITC-MSN** for sensing lysosomal hypochlorous acid (HOCl).^38^ The probe is composed of silica nanoparticles with a fluorescein fluorophore as the donor dye and a non-fluorescent spirothioether unit which reacts with HOCl to generate a fluorescent rhodamine as the acceptor dye. **ThioRB-FITC-MSN** has an emission maximum at *λ*_em_ = 526 nm (*λ*_ex_ = 490 nm). In the presence of HOCl, an increase in emission at *λ*_em_ = 586 nm accompanied by decreased emission at *λ*_em_ = 526 nm occurs, which was attributed to the HOCl-triggered tandem oxidation and β-elimination of the spirothioether-bearing unit to produce rhodamine, facilitating FRET from the fluorescein donor to the rhodamine acceptor and emission at *λ*_em_ = 586 nm (Scheme 2). **ThioRB-FITC-MSN** exhibited good selectivity towards HOCl over other reactive oxygen and nitrogen species (ROS/RNS) including nitric oxide (NO), H_2_O_2_, ˙OH, ROO˙ or O_2_˙^−^. Furthermore, after L929 cells were stained with **ThioRB-FITC-MSN** and LysoTracker Blue DND-22 (a lysosome marker), L929 cells exhibited excellent fluorescence co-localisation of the probe and LysoTracker Blue, which indicated that **ThioRB-FITC-MSN** was suitable for targeting the lysosomes of L929 cells.

**Scheme 3:**
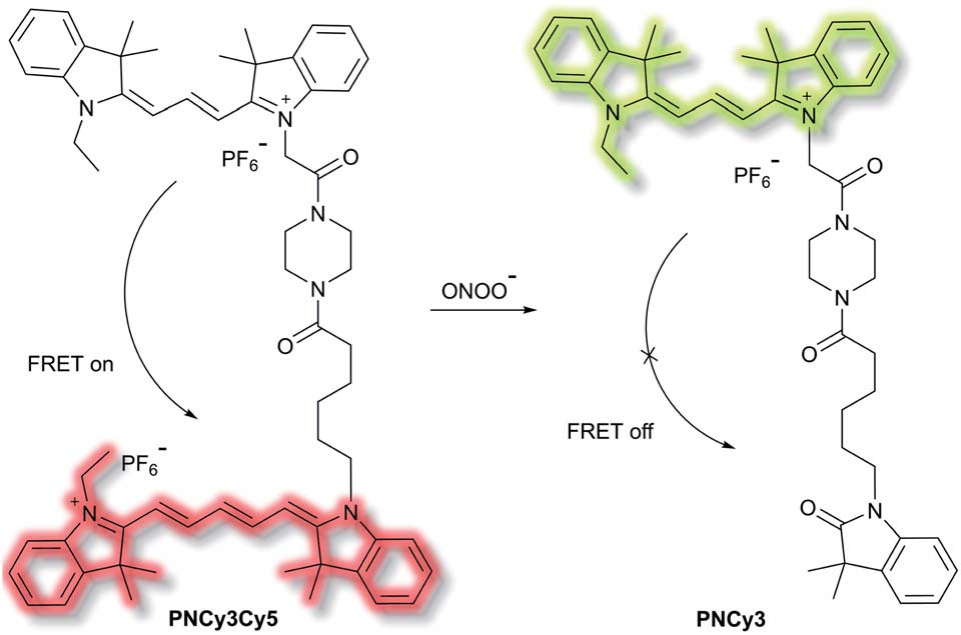
Cy3 and Cy5 (energy donor and acceptor)-based FRET fluorescent probe **PNCy3Cy5**, developed for the ratiometric detection of ONOO^−^. Also shown is the structure of its cleavage product **PNCy3**.

Qian *et al.* have developed a FRET-based small molecule fluorescent probe **PNCy3Cy5** for the selective detection of peroxynitrite (ONOO^−^) in living cells.^39^ The cyanine dyes Cy3 and Cy5 are bright and photostable, and are well suited for the fluorescence imaging of living cells. **PNCy3Cy5** contains a Cy3 and Cy5 dyad which serves as the fluorescence energy donor and acceptor pair separated by an acetyl–piperazinyl–hexanoyl spacer that tethers the Cy3 and Cy5 together (Scheme 3). **PNCy3Cy5** displays fluorescence emission for Cy5 (*λ*_ex_ = 530 nm, *λ*_em_ = 660 nm) due to FRET from Cy3. However, in the presence of ONOO^−^, the Cy5 moiety was selectively oxidised to its oxindole derivative, terminating the FRET process, resulting in a fluorescence increase at *λ*_em_ = 560 nm and a decrease at *λ*_em_ = 660 nm, the fluorescence intensity ratio *I*_560nm_/*I*_660nm_ displaying a nearly 324-fold enhancement. The proposed cleavage-based mechanism of action was supported by mass spectrometric analyses, which confirmed the formation of the oxidized product **PNCy3**. Furthermore, **PNCy3Cy5** exhibits an excellent detection limit of 0.65 nM and high detection selectivity in 0.1 M phosphate buffer (0.2% DMF, v/v, pH = 7.4). Inspired by these results, **PNCy3Cy5** was used as a ratiometric sensor for both exogenous and endogenous fluorescence imaging of ONOO^−^ in RAW264.7 macrophages.

**Scheme 4:**
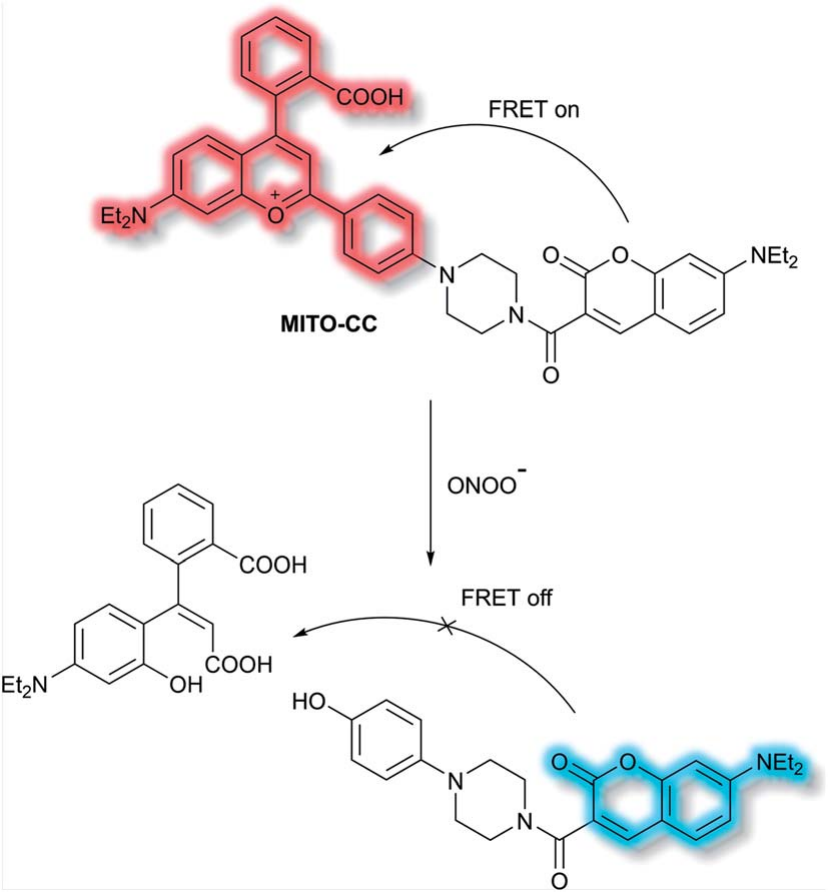
FRET-based ratiometric fluorescent probe **MITO-CC** for the detection of ONOO^−^.

Chang *et al.* have reported a TP ratiometric fluorescent probe **MITO-CC** based on FRET for the detection of mitochondrial ONOO^−^.^40^**MITO-CC** combined a modified chromenylium fluorophore as the energy acceptor and a modified coumarin as the energy donor (Scheme 4). In the absence of ONOO^−^, **MITO-CC** exhibited a strong emission at 651 nm and a weak emission of coumarin at 473 nm. Upon addition of ONOO^−^ (0 to 7.5 μM), a 93-fold fluorescence ratio (*I*_473nm_/*I*_651nm_) enhancement was reported. Furthermore, a low limit of detection (LoD, 11.3 nM), fast response (less than 20 s) and high selectivity over other biological ROS/RNS including NO, NO_2_^−^, H_2_S, H_2_S_2_, HNO, H_2_O_2_ and HOCl in PBS buffer solution (25 mM, containing 30% ethanol, pH 7.4) were observed. Moreover, the low cytotoxicity of **MITO-CC** facilitated the monitoring of endogenous ONOO^−^ in HepG2/RAW264.7 cells and an LPS-stimulated mouse model.

**Scheme 5:**
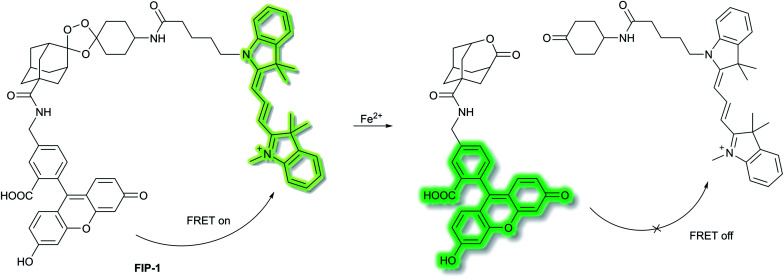
FRET-based ratiometric fluorescent probe **FIP-1** for the detection of Fe^2+^.

Chang *et al.* have developed a novel FRET-based ratiometric fluorescent probe **FIP-1** for the detection of ferrous cation (Fe^2+^) in living systems.^41^**FIP-1** links a 5-aminomethyl fluorescein (5-AMF) donor and a cyanine 3 (Cy3) acceptor through an endoperoxide bridge as a Fe^2+^ responsive trigger (Scheme 5). The initial state of probe **FIP-1** is FRET on, with emissions at 515 nm and 556 nm, when excited at 488 nm. However, with the addition of Fe^2+^ the endoperoxide core can be cleaved, which leads to dissociation of the donor and acceptor, resulting in FRET turn off. This leads to increased emission at 515 nm and loss at emission at 556 nm, corresponding to the emission of the 5-AMF donor. **FIP-1** exhibited high selectivity and sensitivity towards Fe^2+^ over other competing biologically relevant metals such as Fe^3+^, Cu^2+^ and Zn^2+^, facilitating the imaging of labile iron pools in HEK 293T cells.

**Scheme 6:**
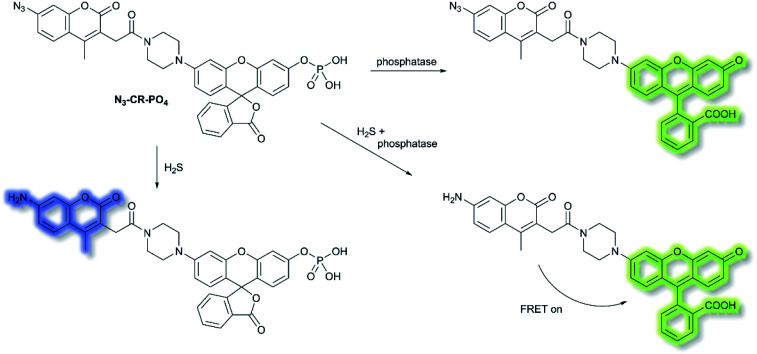
FRET-based **N**_**3**_**-CR-PO**_**4**_ developed for the detection of H_2_S and phosphatase.

Reactive sulfur species (RSS) have become an important research area due to their vital physiological functions. Among the RSS, hydrogen sulfide (H_2_S) is perhaps the most attractive to study, since changes in H_2_S metabolism can lead to an array of pathological disturbances in the form of hypertension, atherosclerosis, heart failure, and diabetes.^42,43^ In 2019, Zhang *et al.* developed a single dual-reactive FRET-based fluorescent probe **N**_**3**_**-CR-PO**_**4**_ for monitoring how phosphatase activity depends on the levels of H_2_S in cells. The FRET system consists of an N_3_-modified coumarin and phosphate-modified rhodol, connected by a piperazine bridge.^44^ The proposed sensing mechanism is depicted in Scheme 6. Initially, **N**_**3**_**-CR-PO**_**4**_ exhibits relatively weak fluorescence peaks at 445 and 545 nm upon excitation at 360 nm in Tris–HCl buffer (pH 7.2). However, upon addition of H_2_S, a blue fluorescence at *λ*_em_ = 445 nm (*λ*_ex_ = 360 nm) was observed due to the reduction of the –N_3_ to –NH_2_. Subsequently, on the addition of alkaline phosphatase (ALP), the phosphate group of **N**_**3**_**-CR-PO**_**4**_ was cleaved, leading to recovery of the green fluorescence at *λ*_em_ = 545 nm (*λ*_ex_ = 510 nm) of rhodol in Tris–HCl buffer (pH 8.0). In the presence of fixed concentrations of H_2_S followed by addition of ALP, the fluorescence at *λ*_em_ = 445 nm decreased gradually, and the fluorescence at *λ*_em_ = 545 nm significantly increased, due to FRET from the donor (coumarin) to the acceptor (rhodol). Furthermore, **N**_**3**_**-CR-PO**_**4**_ was shown to have high selectivity for ALP over other enzymes, amino acids, proteins, peptides, and inorganic salts. Importantly, it was confirmed in HeLa cells that intracellular concentrations of H_2_S exhibited a crucial role in regulating the activity of the phosphatase enzyme. It was found that cells can regulate their H_2_S concentrations in order to maintain an optimal level of phosphatase activity.

**Scheme 7:**
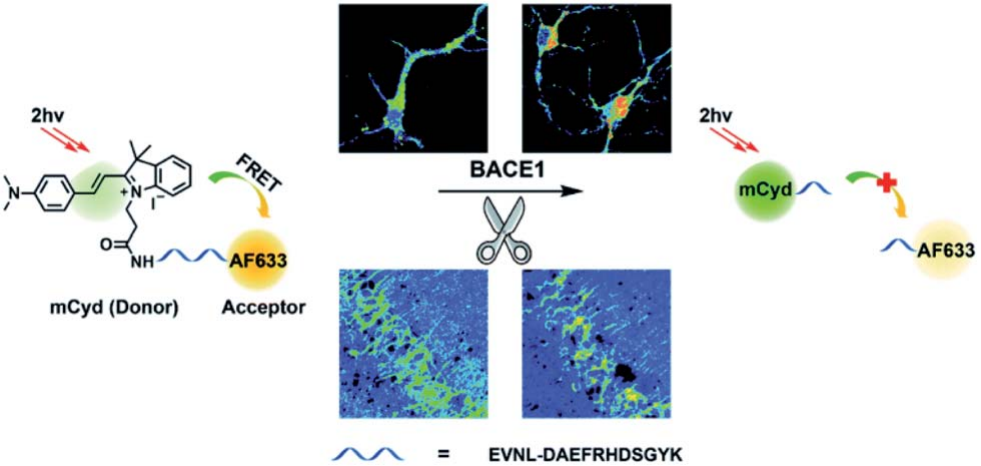
Illustration for the working principle of the designed TP ratiometric fluorescent probe **AF633mCyd**, for the determination of BACE1 in neurons and mouse brain tissue slice. Reproduced with permission from ref. 47. Copyright 2020, The Royal Society of Chemistry.

β-Secretase (BACE1) is an important enzyme in Alzheimer's disease (AD).^45^ β-amyloid (Aβ) peptides are generated by sequential cleavages from BACE1, and the accumulation of Aβ is responsible for the pathogenesis of AD.^46^ Recently, Tian and colleagues have created a FRET-based TP ratiometric fluorescent probe **AF633mCyd** for the bioimaging and sensing of BACE1 in different regions of AD mouse brain. The FRET system contains an acceptor – Alexa Fluor 633 (AF633) and a donor – merocyanine derivative (mCyd) which are connected by a linker that is a substrate of the enzyme, with a chain length less than 10 nm, as shown in Scheme 7.^47^ The peptide substrate (EVNL-DAEFRHDSGYK) was inserted between the donor and acceptor, then because the linker can be specifically cleaved by BACE1 the donor and acceptor are separated. The sensing mechanism was confirmed using mass spectrometry. **AF633mCyd** exhibited a maximum emission at 651 nm upon TP excitation at 820 nm, which was ascribed to the emission profile of the fluorescent acceptor AF633. Exposure of **AF633mCyd** to BACE1 results in the separation of the donor and acceptor in this system which caused a reduction in the AF633-based fluorescence emission at 651 nm and an increase in mCyd-based fluorescence emission at 578 nm. Importantly, **AF633mCyd** displayed high selectivity and sensitivity, as well as good biocompatibility and long-term stability, facilitating its application for the bioimaging and sensing of BACE1 in live neurons and different regions of AD mice brains. The fluorescence emission ratio of **AF633mCyd** displayed good linearity with concentrations of BACE1 from 0.1 to 40.0 nM providing a LoD of 65.3 ± 0.1 pM (fresh cell lysates containing 0.05% DMSO, pH 4.5).

FRET-based fluorescent probes are widely used to monitor cellular dynamics in living cells in real time. Advantages of FRET-based fluorescent probes include large Stokes shifts, ratiometric sensing, and dual/multi-analyte responsive systems.^11^ Despite the value of these characteristics, the applicability of FRET is still dependent on the FRET efficiency; the distance between the donor and acceptor moieties strongly influences the efficiency of FRET. Therefore, this technology still requires further investigation and development. For instance, combining FRET with advanced fluorescence microscopy techniques, such as fluorescence lifetime imaging microscopy (FRET-FLIM), offers a sensitive method for lifetime measurements.^48^

## 3. Intramolecular charge transfer (ICT) probes

A common approach for analyte detection is the use of intramolecular (or internal) charge transfer (ICT) probes. Fluorophores of ICT probes are made of 3 conjugated parts – an electron donor (D), an electron acceptor (A) and π-conjugated linker.^1^ When ICT probes interact with the desired analyte, the electron density in the recognition group is altered (*e.g.* as a result of bond cleavage, substitution, or substrate coordination) which installs a ‘push–pull’ system in the molecule. Excitation of the resultant species, in tandem with the altered electron distribution, affords a large dipole and altered excited state to that of the initial probe.^49^ As such, ICT probes commonly exhibit a ‘turn-on’ fluorescence response and wavelength shift of the fluorescence after interaction/recognition of the analyte (though there key instances where ICT probes allow for a ratiometric analysis, where instead a shift of the fluorescence response is given).^50,51^

**Scheme 8:**
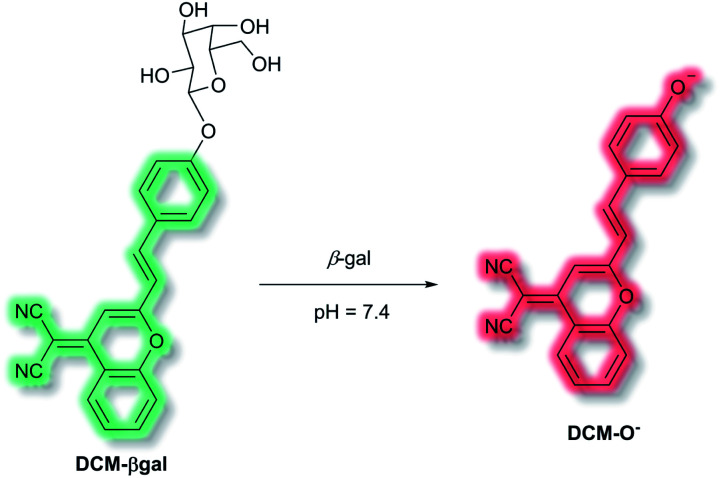
Detection mechanism of probe **DCM-βgal** towards *β*-gal.

Use of ICT probes is particularly prevalent for detecting enzyme activity, especially when coupled with the advantages of NIR fluorescence (*e.g.* deep tissue penetration, low background fluorescence).^52^ In 2016, Zhu *et al.* reported an *in vivo* NIR probe to target enzyme *β*-galactosidase (*β*-gal), a biomarker for cell senescence and primary ovarian cancers.^53^ The probe, **DCM-βgal**, was composed of a dicyanomethylene-4*H*-pyran (**DCM**) NIR fluorophore connected to a sugar recognition moiety. Initial evaluation using PBS/DMSO indicated that in the presence of *β*-gal, the sugar moiety of **DCM-βgal** could be cleaved to release **DCM-O−** (Scheme 8). The process was found produce a ratiometric (*λ*_ex_ = 450 nm, *λ*_em_ = 685 nm and 500 nm) and turn-on (*λ*_ex_ = 535 nm, *λ*_em_ = 685 nm) fluorescence response depending on the excitation wavelength. The response of **DCM-βgal** towards *β*-gal was rapid, with a plateau in fluorescence after 35 minutes. A detection limit of 1.7 × 10^−4^ U mL^−1^ was obtained. **DCM-βgal** was highly selective towards *β*-gal over other enzymes (cellulase, reductase, lysozyme, esterase) and biologically relevant species (Cys, Hcy, GSH, H_2_O_2_, H_2_S, dithiothreitol). **DCM-βgal** could be used to visualise 293T (human embryonic kidney) cells. Specifically, 293T cells with the *lacZ* gene (*i.e.*, over expressing *β*-gal) resulted in a fluorescence decrease of the green channel (*λ*_em_ = 490–530 nm) and increase in the red channel (*λ*_em_ = 650–720 nm). Conversely, 293T cells lacking *lacZ* displayed fluorescence predominantly in the green channel over the red channel. A 5-fold increase in the ratiometric fluorescent response was measured when the *lacZ* gene was present. Finally, **DCM-βgal** was evaluated in tumour-bearing mice; fluorescence was observed within 5 minutes of **DCM-βgal** injection. This work reports the first real-time *in vivo* 3D imaging of *β*-gal.

**Scheme 9:**
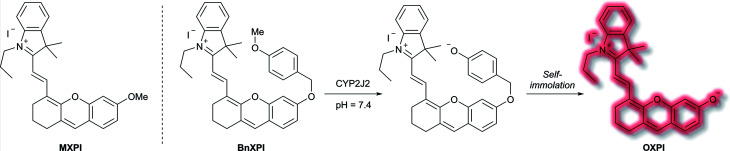
Mechanism of detection of CYP2J2 by probe **BnXPI**, with initial test compound **MXPI** and fluorophore **OXPI** (deprotonated **HXPI**) also shown.

A family of enzymes that have received significant attention are the cytochrome P450 (CYP) enzymes.^54,55^ In 2019, Ma *et al.* described a probe to determine the catalytic activity of CYP450, and in particular the isoform CYP2J2, a potential cancer biomarker involved in cell proliferation, angiogenesis, and metastasis.^56,57^ Like CYP1A, CYP2J2 effects *o*-dealkylation of its substrates. The **HXPI** fluorophore was selected based on its good tissue penetration, low cytotoxicity, and the flexibility of the molecule favouring binding in the active site of CYP2J2. Initial screening of the recognition moieties indicated that *o*-methylated **HXPI**, **MXPI**, was able to fit the active site of CYP2J2, but metabolism of the recognition group was low. Addition of the self-immolative *p*-hydroxybenzyl (PHB) linker was found to increase metabolism of the probe. *In silico* studies rationalised this as being due to the placement of the OMe group closer to the catalytic site of CYP2J2. Screening a series of *o*-alkyl recognition moieties found that *o*-methylation was optimal, overall affording **BnXPI** (Scheme 9). In PBS solution (pH 7.4), **BnXPI** exhibited a turn-on fluorescent response towards CYP2J2 (*λ*_ex_ = 656 nm, *λ*_em_ = 718 nm), and was found to be highly selective (46-fold) towards CYP2J2 (detection limit of 0.024 mg mL^−1^) when compared to other CYP isoforms. Testing of **BnXPI** in HLMs showed that the probe could successfully monitor CYP2J2 activity with calculated metabolic rates comparable to those of determined using the nonfluorescent standard astemizole. NIR fluorescence bioimaging of CYP2J2 in cells was achieved in a range of human cancer cell lines (U251, HepG2, A549, and K562) within 1 h at physiological pH (*λ*_ex_ = 633 nm, *λ*_em_ = 690–750 nm). Exogenous inhibition of CYP2J2 confirmed the observed fluorescent enhancements were afforded by CYP2J2. Furthermore, **BnXPI** was shown to detect angiogenesis in human umbilical vein endothelial cells (HUVEC) *in vivo* and *ex vivo*; significant fluorescent enhancement was achieved and neovessel growth could be followed due to the increased expression of CYP2J2 in such environments. In addition, **BnXPI** could be used as a clinical tool for patients with cancer. In healthy patients, peripheral blood samples treated with **BnXPI** showed little fluorescence response. Conversely, samples taken from lymphoma and leukaemia patients showed significant fluorescent turn-on response, which was found to be proportional to expression of CYP2J2. Finally, it was shown that **BnXPI** could be used for real-time *in vivo* imaging of tumours in mice. Injection with **BnXPI** afforded both a rapid (<10 min) and selective fluorescence response localised within the tumour region.

**Scheme 10:**
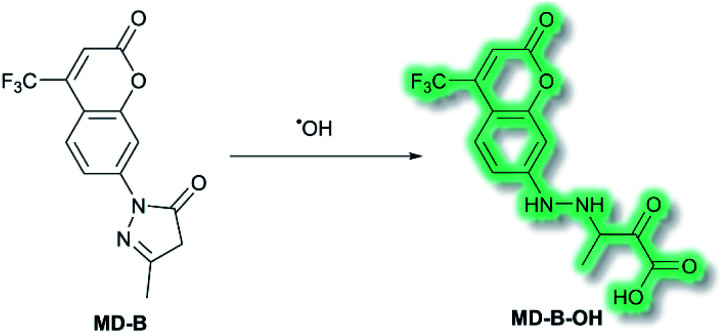
Detection of ˙OH with ICT probe **MD-B**.

Another area in which NIR ICT probes have been used is in the detection of (non-peptidic) biologically relevant small molecules. One such class of analytes are reactive oxygen species (ROS), which are associated with oxidative stress and as a result received much attention in the world of fluorescent probes for biological applications.^58,59^ In 2019, a TP fluorescent probe for the detection of the ROS hydroxyl radical (˙OH) was developed by Tang *et al.*^60^ It is known that ˙OH can cause oxidative damage, which has been suggested to contribute to the pathogenesis of depression. The authors proposed that live monitoring of ˙OH in the brain is key to understanding this relationship, and of all the bioimaging tools two photon microscopy (TPM) would be the most advantageous. Probe **MD-B** incorporated a courmarin 151 (Cou151) fluorophore due to its known TP properties, large Stokes shift, and ICT character. A pyrazolone was employed as a recognition group for ˙OH, in which the electron donor ability of the anilinic nitrogen incorporated into the pyrazolone system is attenuated, thereby masking the inherent ICT system of Cou151. The initial OP (*λ*_ex_ = 370 nm, *λ*_em_ = 500 nm) and TP (*λ*_ex_ = 800 nm, *λ*_em_ = 500 nm) experiments in PBS-buffer (pH 7.4) indicated that **MD-B** exhibits a significant turn-on fluorescence response, which scaled linearly with ˙OH. It was proposed that the turn-on response upon addition of ˙OH was caused by a single-electron oxidation of **MD-B** to form fluorescent species **MD-B-OH**, in which the Cou151 ICT system is restored (Scheme 10). An increase in quantum yield (*φ*_f_ = 0.037 to *φ*_f_ = 0.25 for **MD-B** and **MD-B-OH** respectively) and in TP action cross section (*σ* = 5.0 GM to *σ* = 42.6 GM) was observed. **MD-B** was also found to selectively detect ˙OH over all other ROS tested, including O_2_˙^−^, H_2_O_2_, TBHP, OCl^−^, ^1^O_2_, NO, ONOO^−^, 2,2′-azobisisobutyronitrile (AIBN), 2,2′-azobis(2,4-dimethylvaleronitrile) (AMVN), and CO_3_˙^−^. A detection limit for **MD-B** towards ˙OH was determined as 2.4 nM. **MD-B** was successfully employed to detect ˙OH in human astrocytes pre-treated with the ˙OH inducer glutamate, in PC12 cells, and in mouse RAW 264.7 cells by TP fluorescence (*λ*_em_ = 400–650 nm). Furthermore, human astrocytes treated with the ˙OH scavenger mannitol, as well as glutamate, saw a significantly decreased fluorescence response. **MD-B** was shown to be able to cross the blood–brain barrier and to detect ˙OH in the brains of mice under the stress of restraint. Furthermore, **MD-B** could actively monitor fluctuating levels of ˙OH in mice with depression-like behaviour – mice exposed to chronic unpredictable mild stress (CUMS). Remarkably, TP images of the brains of CUMS mice indicated a significant (6-fold) turn-on fluorescence in contrast to control mice. It was hypothesised that **MD-B** could be used to evaluate the connection between ˙OH and SIRT1, an acetylase enzyme associated with depression. Through a combination of ˙OH inhibition and induction experiments in human astrocytes, it was confirmed that SIRT1 activity was reduced by ˙OH. Furthermore, LC-MS/MS proteomic analysis indicated that ˙OH oxidises the phenylalanine residues in the active site of SIRT1, thereby reducing its activity. From these results the authors suggested that ˙OH, due to its role in stress and SIRT1, must play an important role in depression.

**Scheme 11:**
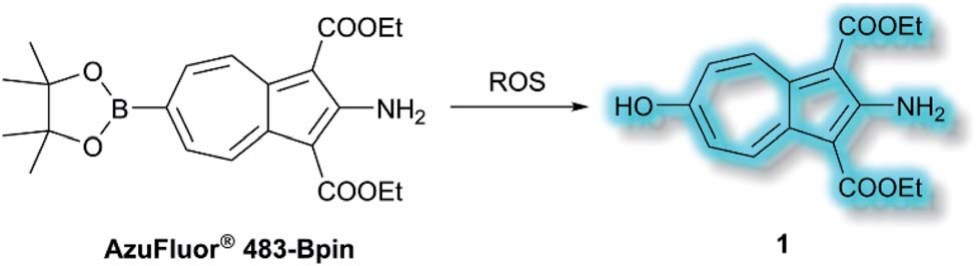
Detection of ROS with TP ICT probe **AzuFluor® 483-Bpin**.

Also in 2019, Lewis *et al.* reported an azulene probe for detection and imaging of ROS, the first probe employing an azulene fluorophore for fluorescence microscopy.^61^ Here, the Bpin group was chosen as the receptor motif due to its high affinity and selectivity for ONOO^−^.^62^ It was proposed that upon addition of ONOO^−^, and reaction to give the corresponding alcohol, the electron-donating –OH group would reinforce the inherent polarity of the azulene core *via* an ICT mechanism and in doing so cause a turn-on fluorescence response. Ethyl esters were incorporated at the 1- and 3-positions to increase the stability of the probe, overall affording **AzuFluor® 483-Bpin**. Upon reaction with H_2_O_2_, **AzuFluor® 483-Bpin** was rapidly converted to 6-hydroxy species **1** (Scheme 11). As expected, **AzuFluor® 483-Bpin**, was found to be non-fluorescent, but underwent a significant fluorescent turn-on in the presence of ROS as **1** is formed (*λ*_ex_ = 350 nm, *λ*_em_ = 483 nm). As expected **AzuFluor® 483-Bpin** was found to be highly selective towards ONOO^−^ over other ROS at equivalent concentrations, but also exhibited a response to H_2_O_2_ at higher concentrations. Emission at *λ*_em_ = 483 nm was enhanced with increasing [ONOO^−^] or [H_2_O_2_], with detection limits calculated to be 21.7 nM and 1.72 μM respectively. **AzuFluor® 483-Bpin** was then evaluated as a TP chemodosimeter for ROS. The maximum TP fluorescence emission was observed at 700 nm for **AzuFluor® 483-Bpin**, which upon addition of ONOO^−^ was found to shift to 810 nm. At 810 nm, the TP action cross-section of **1** was found to be *σ* = 3.2 GM. *In vitro* HeLa cell studies indicated that when excited at 800 nm, the emission intensity of **AzuFluor® 483-Bpin** + ONOO^−^ increased 4-fold in respect to **AzuFluor® 483-Bpin**. The probe was also shown to successfully undergo turn on fluorescence, and subsequent TPM imaging, of RAW 264.7 cells pre-treated with inducers of endogenous ROS. Both induction and inhibition studies confirmed the fluorescence response was due to the interaction of **AzuFluor® 483-Bpin** and ROS. Finally, **AzuFluor® 483-Bpin** was used to successfully image endogenous ONOO^−^ and H_2_O_2_ in rat hippocampal tissue *via* TP microscopy (TPM, *λ*_ex_ = 800 nm, *λ*_em_ = 400–600 nm), demonstrating that **AzuFluor® 483-Bpin** can detect ROS in tissue.

A class of biomolecules often studied in tandem with ROS are biothiols, which commonly act as antioxidants in many biological systems, as well as being associated with various disease states (*e.g.*, Alzheimer's disease, liver damage). In 2016, Lin *et al.* published an NIR probe able to detect and image biothiols at both high and low concentrations.^63^ It was proposed that use of a single probe with 2 distinct chemical handles would allow for the detection of multiple biothiol species that would otherwise require multiple probes, avoiding any associated issues (*e.g.* fluorescence cross talk, localisation issues). The probe **CHMC-Thiol** consisted of chlorohydroxylmerocyanine (**CHMC**) NIR fluorophore and uses two thiol recognition units. Each receptor has either ‘high-sensitivity’ (2,4-dinitrobenzenesulfonate) or ‘low-sensitivity’ (chloride) towards thiols.

**Scheme 12:**
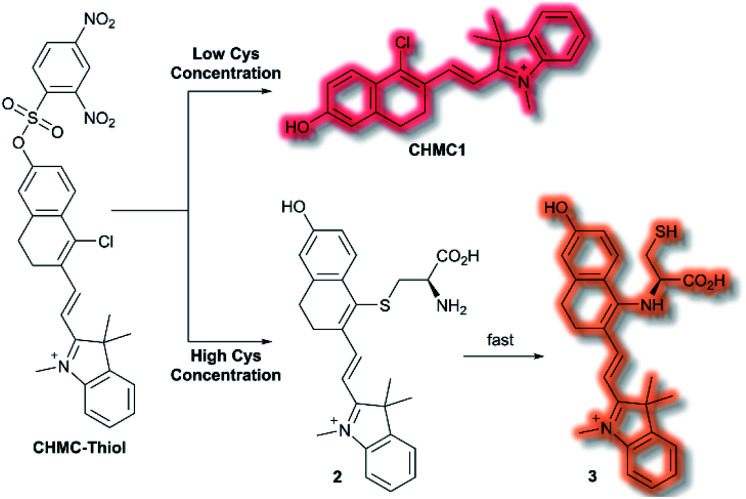
High and low concentration of Cys detection pathways of probe **CHMC-Thiol**.

**CHMC-Thiol** is non-fluorescent, but upon addition of low concentrations of Cys (0–50 μM, 0–10 eq.) a turn-on fluorescence response was observed (*λ*_ex_ = 550 nm, *λ*_em_ = 680 nm). When **CHMC-Thiol** was combined with increasing concentrations of Cys (50–500 μM, 10–100 eq.) a ratiometric fluorescent response was observed (*λ*_em_ = *I*_625nm_/*I*_680nm_). It was proposed that at low concentrations, Cys attacks the **CHMC-Thiol** at the ‘high-sensitivity’ sulfonate group to generate the corresponding fluorescent species **CHMC1**, in which the liberated phenol moiety creates an ICT system (Scheme 12). Reaction of **CHMC-Thiol** with higher concentrations of Cys effects both cleavage of the ‘high-sensitivity’ recognition moiety, and in addition substitution of the ‘low-sensitivity’ chloro group attached to the **CHMC** core. Initial attack of the thiol in Cys generates intermediate **2** in which Cys is S-linked to the **CHMC** core, which undergoes an intramolecular addition–elimination reaction to afford N-linked **3**. **CHMC-Thiol** could be used to detect Hcy and GSH. **CHMC-Thiol** reacts with Hcy to induce a fluorescence response analogous to the reaction with Cys. While with GSH, at low concentrations a turn-on fluorescence at *λ*_em_ = 680 nm was observed, but at higher concentrations only the thioether product was formed, and no intramolecular addition/elimination occurs. This resulted in a 10 nm redshift (*λ*_em_ = 690 nm) in fluorescence emission. The selectivity of **CHMC-Thiol** at both *λ*_em_ = 680 nm and *λ*_em_ = 625 nm was determined. As expected, the probe exhibited a response towards GSH, Hcy, and Cys at 680 nm. A mild response was also elicited with Na_2_S. All other analytes tested (metal cations, ROS, RNS, and biological anions) did not generate a fluorescence response. Conversely, at 625 nm a fluorescent response was only observed for Cys and Hcy. **CHMC-Thiol** was then evaluated in living cells. A strong fluorescence response was observed in the red channel (*λ*_em_ = 650–750 nm) for HeLa cells incubated with **CHMC-Thiol**. Furthermore, cells treated with exogenous Cys exhibited significant fluorescence enhancement in the orange channel (*λ*_em_ = 580–640 nm), as well as fluorescence in the red channel. From these observations, the authors concluded that **CHMC-Thiol** gave a turn-on response at low Cys concentrations, and a ratiometric response at high Cys concentrations. Analogous results were obtained from imaging experiments using Kunming mice.

**Scheme 13:**
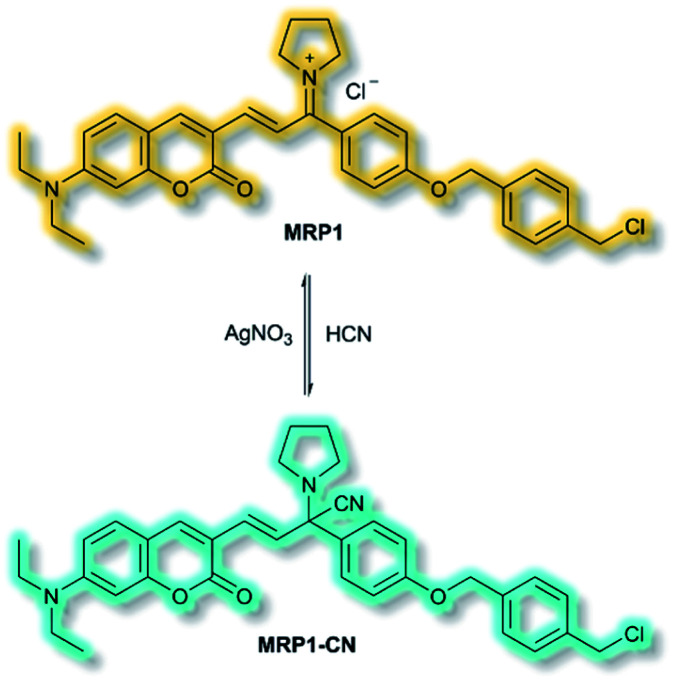
Reversible detection of HCN of ICT probe **MRP1**.

The final probe to be discussed in this section is the mitochondria-specific HCN probe developed by Sessler *et al.* in 2018.^64^ Whilst HCN is a well-recognized toxin, endogenous HCN is thought to play a physiological role within neurons. The probe **MRP1** was designed using the diethylaminocoumarin ICT fluorophore and a methylenepyrrolidinium recognition unit. Addition of a *para*-alkoxy group increased the *φ*_f_ and a benzyl chloride group was included to immobilise **MRP1** within mitochondria by reacting with protein thiols. The recognition unit of **MRP1** was found to react with HCN both reversibly and rapidly to give **MRP1-CN**, exploiting the nucleophilicity of the cyanide anion at physiological pH (Scheme 13). In solution (PBS/MeCN, 1 : 3, v/v, pH 7.4), **MRP1** exhibited an a linear, ratiometric fluorescence response (*λ*_ex_ = 467 nm, *λ*_em_ = *I*_490nm_/*I*_599nm_) upon addition of cyanide, and a detection limit of 65.6 nM. The probe was stable for over 24 h in solution, whilst time trial experiments indicated the detection of KCN in <1 s. **MRP1** was selective towards cyanide over other relevant anions (*e.g.* halides, acetate, sulfate, nitrate, perchlorate and azide). Furthermore, the probe could be cycled ‘on’ and ‘off’ repeatedly by sequential addition of cyanide and AgNO_3_ respectively.

Colocalization experiments with Mitotracker Deep Red indicated that **MRP1** localised in cell mitochondria, with a Pearson's colocalization coefficient of 0.94. Importantly, it was retained in mitochondria after the membrane potential was destroyed. HepG2 cells incubated with only **MRP1** gave no fluorescence in the green channel (*λ*_em_ = 460–530 nm), but strong emission in the red channel (*λ*_em_ = 575–650 nm). Upon addition of KCN, fluorescence enhancement of the green channel was observed alongside reduced emission in the red channel. The green-to-red ratiometric response scaled linearly when KCN was added. Endogenous HCN studies were performed with neuron-like rat pheochromocytoma cells due to their natural production of HCN. Within 15 minutes of addition of **MRP1**, a strong emission was observed in the green channel and weak emission in the red channel. A combination of cyanide stimulation with hydromorphine and quenching with methemoglobin confirmed that the ratiometric response was due to endogenous HCN production. Sequential use of hydromorphine and methemoglobin indicated that **MRP1** was able to dynamically respond to changing HCN concentrations. It should be noted that this is the first example of a fluorescent HCN probe capable of monitoring endogenous HCN.

ICT probes are often simple in design, allowing for straightforward syntheses and clear mechanisms of action upon formation of the D–π–A system. Once a clear a mechanism of detection is established (*e.g.* a turn-on fluorescence response or a ratiometric response), the recognition moieties of ICT probes can often be easily substituted for those that detect other species of interest. As such, ICT systems often provide straightforward routes to a family of probes for an array of analytes. The main limitations of ICT probes are due to the risk of reduced fluorescence as a result of aggregate-caused quenching (ACQ), and that the nature of their fluorescence is dependent on the solvent in which the experiments are performed (due to the corresponding solute–solvate interactions).^65^ The second of these issues can often lead to problems when results from initial organic-solvent based experiments are not reproducible in *in vitro* studies in biological media (*i.e.* buffered aqueous solutions). Furthermore, the broad emissions often exhibited by ICT fluorophores can prove troublesome when the fluorophore exhibits a ratiometric response.^66^

## 4. Photoinduced electron transfer (PeT) probes

**Scheme 14:**
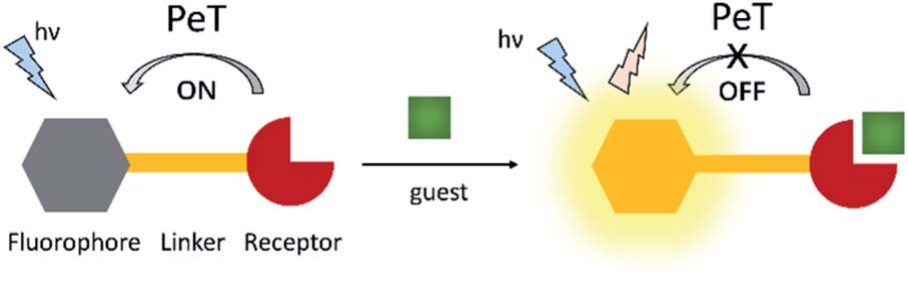
The mechanism of PeT-based fluorescent probes.

Photoinduced electron transfer (PeT) is one of the most important mechanisms for developing fluorescent probes and biosensors. The phenomenon of PeT was first described in the 1970s.^67,68^ With PeT systems, the intramolecular transfer of electrons from the receptor to the fluorophore results in fluorescence quenching. However, when the receptor binds to its target, the PeT process is inhibited or completely suppressed, which means the sensor restores its fluorescence (Scheme 14).^69^ PeT based fluorescence probes usually have “turn on” features, which are conducive to fluorescence imaging with high signal-to-noise ratios.

**Scheme 15:**
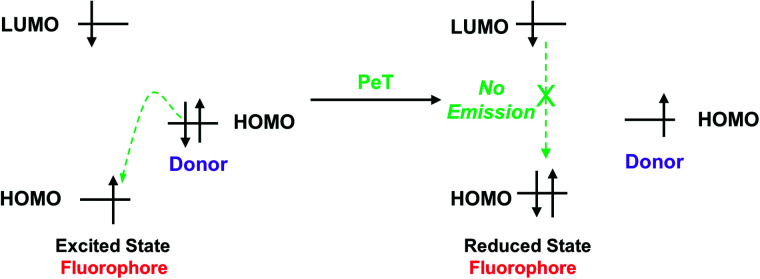
Frontier orbital explanation for the PeT effect.

When a fluorophore is excited by an appropriate wavelength of light, an electron from the highest occupied molecular orbital (HOMO) is transferred to the lowest unoccupied molecular orbital (LUMO). If the energy level of the HOMO of the adjacent receptor group lies between the LUMO and HOMO of the fluorophore, an electron transfers from the HOMO of the receptor to the HOMO of the fluorophore by the action of PeT, “locking” an electron in the LUMO and quenching the fluorescence.^70^ Alternatively, when the receptor binds to a target, the energy level of the receptor HOMO is lowered below that of the HOMO of the fluorophore. As such, the PeT process is blocked, and the fluorescence is restored (Scheme 15).

**Scheme 16:**
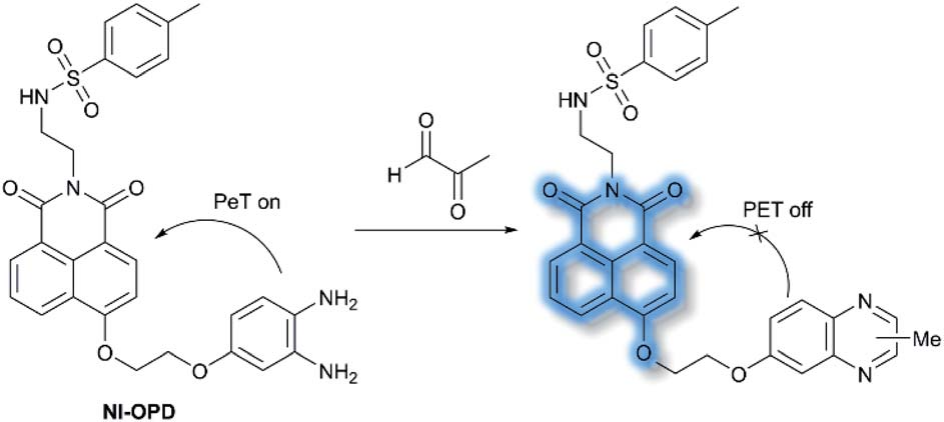
The recognition mechanism of probe **NI-OPD** towards MGO.

In 2018, Peng *et al.* reported a PeT-based endoplasmic reticulum (ER) targeting TP fluorescent probe **NI-OPD**, for detecting methylglyoxal (MGO),^71^ which is associated with diabetes and related complications (Scheme 16).^72^**NI-OPD** contains a 1,8-naphthalimide fluorophore, and *o*-phenylenediamine (OPD) as the MGO recognition unit, with an additional methyl sulfonamide unit as an ER-targeting group. **NI-OPD** was non-fluorescent in PBS buffer (10% DMF, pH = 7.4) due to an “enhanced PeT” process from the OPD unit to the 1,8-naphthalimide moiety. Upon the addition of MGO, the dicarbonyl group of MGO reacted with the OPD group to form a stable adduct that inhibits PeT and affords a turn-on fluorescence (75-fold) at *λ*_em_ = 460 nm (*λ*_ex_ = 380 nm). In addition, **NI-OPD** displayed excellent selectivity toward MGO over other aldehydes, including formaldehyde, acetaldehyde, benzaldehyde, glutaraldehyde, glyoxylic acid and *o*-phthalaldehyde (OPA). Furthermore, **NI-OPD** was evaluated in normal, diabetic and metformin treated diabetic mice to evaluate the difference in their endogenous MGO concentrations. The research indicated that **NI-OPD** could serve as a useful tool for evaluating the involvement of MGO in ER-associated diseases.

**Scheme 17:**
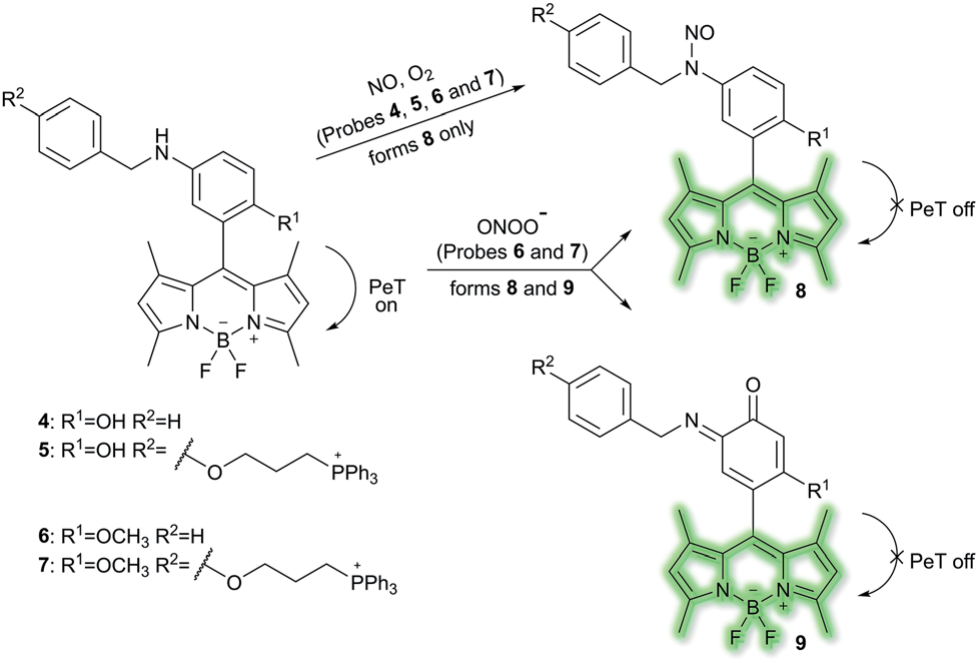
The structures of fluorescent probes **4**, **5**, **6**, and **7**.

Nitric oxide (NO) is a ubiquitous biological messenger, and plays an active role in the regulation of various physiological processes in many biological systems, such as the cardiovascular, immune, and the central and peripheral nervous systems.^73,74^ In 2016, Guo *et al.* developed two fluorescent PeT based NO probes **4** and **5**, where an *N*-benzyl-4-hydroxyaniline group was attached to a BODIPY fluorophore and used as the NO recognition group (Scheme 17).^75^ Probe **4** exhibited almost no fluorescence in PBS buffer (20% MeCN, pH 7.4) due to PeT from the *N*-benzyl-4-hydroxyaniline group to the excited BODIPY dye. However, under aerobic conditions, upon addition of NO, PeT was inhibited affording a dramatic fluorescence enhancement (92-fold) at *λ*_em_ = 518 nm (*λ*_ex_ = 490 nm). The introduction of a triphenylphosphonium (TPP) group within probe **4** afforded the mitochondrial targeting probe **5**, which could specifically accumulate in the mitochondria of HeLa cells and was used to image exogenous NO. Guo and co-workers have also developed a similar probe **6** (Scheme 17),^76^ which exhibited a selective fluorescence off–on response towards NO and ONOO^−^ using *N*-benzyl-4-methoxyaniline as the reaction site rather than the *N*-benzyl-4-hydroxyaniline group. The results indicated that probe **6** exhibits a lower LoD (as low as 0.4 nM) and higher selectivity for NO than probe **4**. Moreover, probe **6** could react with ONOO^−^ to produce an *o*-benzoquinone imine and induced significant fluorescence enhancement. Probe **6** displayed a significant fluorescence off–on response for both NO and ONOO^−^ in HeLa cells. Furthermore, mitochondria-targetable derivative probe **7** was developed by attaching a triphenylphosphonium group to probe **6**, and was used to detect NO and ONOO^−^ in the mitochondria of HeLa cells.

**Scheme 18:**
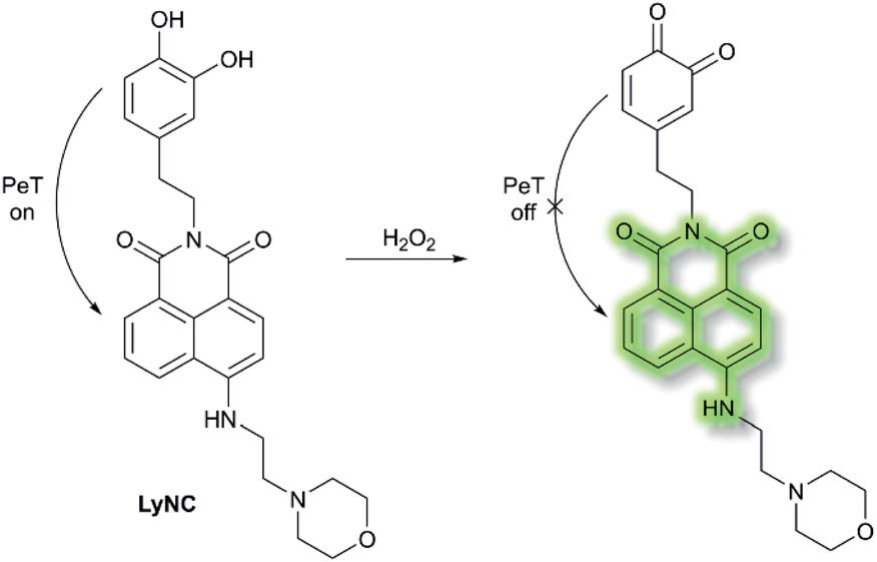
The recognition mechanism of probe **LyNC** towards H_2_O_2_.

Hydrogen peroxide (H_2_O_2_) plays an important role in cell growth, proliferation, host defence, immune response, and signalling pathways under physiological conditions.^77^ In 2017, Kumar *et al.* reported a lysosome targeting PeT-based fluorescence probe **LyNC** for the detection of endogenous H_2_O_2_ in C6 and BV-2 cellular systems.^78^**LyNC** was constructed using a catechol unit as response site for H_2_O_2_ and a morpholine moiety as a lysosome targeting group (Scheme 18). **LyNC** exhibited weak fluorescence in aqueous buffer (0.5% DMSO/PBS, pH 7.4), due to the PeT effect from the catechol to the naphthalimide fluorophore. However, in the presence of H_2_O_2_, the catechol was oxidised to an *o*-quinone, which inhibited PeT thereby facilitating a fluorescence enhancement at *λ*_em_ = 537 nm (*λ*_ex_ = 450 nm). Furthermore, **LyNC** exhibited high sensitivity toward H_2_O_2_ with a LoD of 0.22 μM, and excellent selectivity over other ROS/RNS and biothiols including cysteine, glutathione and homocysteine. Importantly, **LyNC** has been successfully used to detect H_2_O_2_ and in rat brain tissues and in living nematodes.

**Fig. 2:**
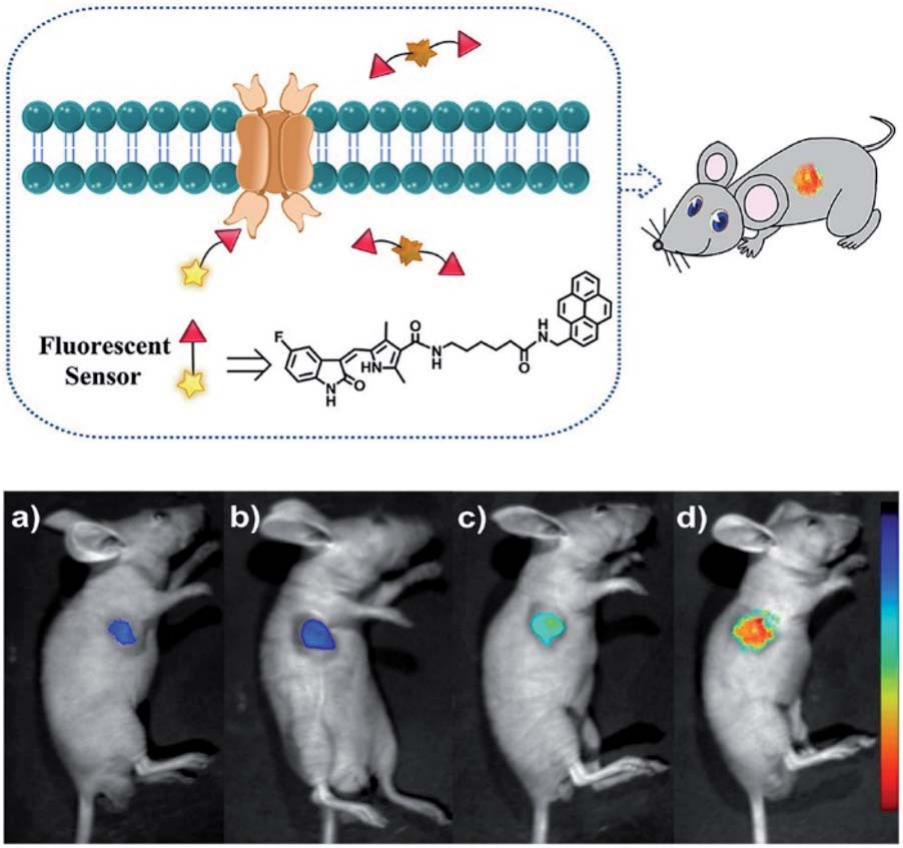
Schematic diagram of **SP1** and *in vivo* fluorescence imaging of **SP1** in a HT-29 tumour-bearing mouse model *via***SP1** injection: (a) 0.1 mM, 100 μL; (b) 0.5 mM, 100 μL; (c) 1 mM, 100 μL; (d) 2 mM, 100 μL. The fluorescence signal was imaged at 500 to 720 nm under excitation with a 460 nm CW laser (power density of 1 mW cm^−2^). Reproduced with permission from ref. 79. Copyright 2018 American Chemical Society.

Duan *et al.* have developed a receptor-targeted fluorescent “off–on” probe **SP1** for the detection and imaging of receptor protein–tyrosine kinases *in vivo* and *in vitro*.^79^**SP1** was composed of a sunitinib targeting group, a six-carbon bis(amide) linker and a pyrene fluorophore. The authors propose that the fluorescence of **SP1** is quenched by dimer formation and π–π stacking interactions forming between the pyrene units and the fluorescence can be restored by binding of the sunitinib group with receptor protein–tyrosine kinases (Fig. 2). Initially, **SP1** displayed weak emission at *λ*_em_ = 545 nm (*λ*_ex_ = 460 nm) in a DMSO/H_2_O simulated physiological medium. In the presence of the protein–tyrosine kinase receptor, an “off–on” fluorescence change was observed. **SP1** was highly selective towards protein–tyrosine kinase receptor over other amino acids, inorganic salts, and other relevant substances. More importantly, **SP1** was used to image tyrosine kinase in chick embryo chorioallantoic membrane and a HT-29 tumour-bearing mouse model, indicating that it could be used for real-time visualisation of tyrosine kinases in tumours.

By using PeT-based fluorescent probes, the localization, distribution and conformational changes of target molecules can be easily unveiled. When compared to sensors without fluorescence switches, PeT-based probes can easily achieve high signal-to-noise ratios, which is an essential requirement for satisfactory *in vitro* and *in vivo* fluorescence imaging. In addition, fluorescent prodrugs based on PET mechanisms are suitable agents for cancer diagnosis and therapy.^80^ As such, the precise regulation of PeT processes is an avenue of development for the future improvement of PeT based prodrugs, which could improve the therapeutic effects and reduce the side effects in cancer treatment. However, a common disadvantage of PeT-based probes is the interference of protons, which also bind with the coordination site, inhibiting the PeT process, and enhancing the fluorescence.^81^ While, the development of PeT probes with lower p*K*_a_ values can offer a promising pathway for eliminating such proton interference.

## 5. Excited state intramolecular proton transfer (ESIPT) probes

**Scheme 19:**
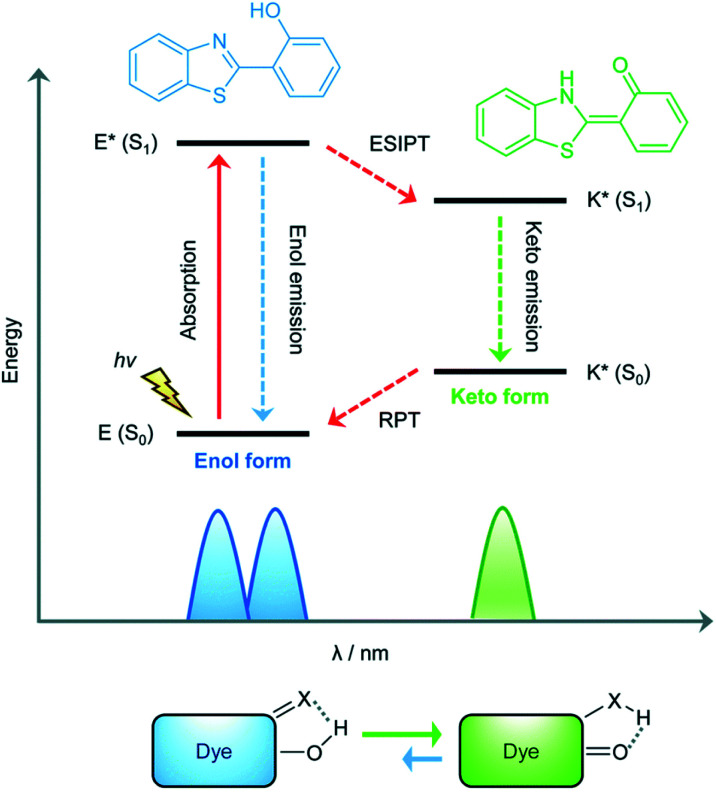
Diagrammatic description of the **ESIPT** process. Reproduced with permission from ref. 13. Copyright 2018, The Royal Society of Chemistry.

The excited state intramolecular proton transfer (ESIPT) process was first reported by Weller in the 1950s.^82^ In general, ESIPT refers to the transfer of hydrogen atoms (mainly from hydroxyl or amino groups) to nearby heteroatoms (mainly N, O or S) under excitation.^83^ Fluorophores with ESIPT properties can establish a fast four-level photocycle between keto and enol isomers under excitation.^13^ ESIPT fluorophores, in the electronic ground state, usually exist in the enol (E) form. Upon photoexcitation, the electronic charge of the molecule is redistributed, leading to greater acidity of the hydrogen bond donor group and increased basicity of the hydrogen bond acceptor within the E form. Therefore, an extremely fast enol to keto phototautomerization (*K*_ESIPT_ > 10^12^ s^−1^) occurs: the excited state enol (E*) is rapidly converted to the excited ketone (K*). After a radiative decay pathway to the electronic ground state, a reverse proton transfer (RPT) takes place to produce the original E form (Scheme 19). The remarkable characteristics of ESIPT fluorophores are: large Stokes shift,^84^ and dual emission,^85,86^ which make them attractive units for the construction of functional fluorescent probes.

**Scheme 20:**
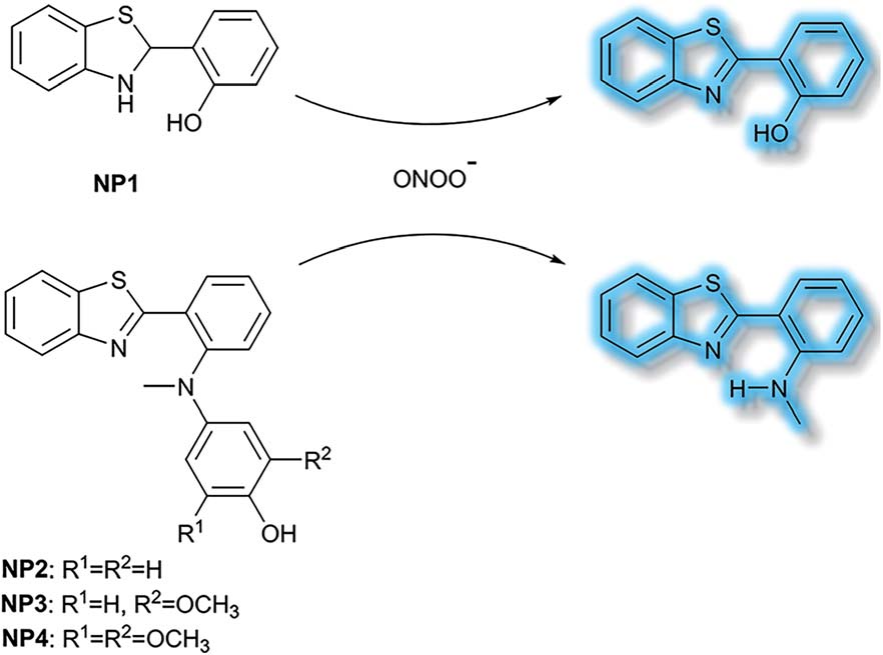
The structures of fluorescent probes **NP1**, **NP2**, **NP3** and **NP4**.

Li *et al.* have developed a series of 2-(2-hydroxyphenyl)benzothiazole-based (HBT-based) ESIPT probes **NP1**, **NP2**, **NP3** and **NP4** for the detection of ONOO^−^ with good specificity, fast response time, and high sensitivity (Scheme 20).^87^**NP1** responds rapidly to ONOO^−^ producing fluorescence enhancements in PBS (pH 7.4), however, it was found to be unstable when exposed to air, therefore, in order to improve molecular stability, **NP2**, **NP3** and **NP4** were developed. The hydroxyl group of HBT was switched to a *N*-methyl-*p*-hydroxyaniline, which blocked the hydrogen donor of the ESIPT process, and electron-donating groups (–OCH_3_) were added to the *p*-hydroxyaniline ring in order to make the hydroxyl group more susceptible to oxidation. Exposure of **NP3** to ONOO^−^ results in the phenol group oxidizing to a benzoquinone imine, accompanied by N–C(sp^2^) bond cleavage, leading to a significant increase in the fluorescence intensity (600-fold). Moreover, **NP3** was capable of crossing the blood–brain barrier (BBB) and could be used for TP fluorescence spectroscopy. The maximal TP absorption cross section of probe **NP3** was determined to be 3.6 GM at 820 nm. This enabled probe **NP3** to be used to visualize ONOO^−^ in neurovascular ischemia progression in the brains of live mice. The excellent properties of **NP3** indicate that it could be used for visualizing other pathophysiological processes associated with ONOO^−^.

**Scheme 21:**
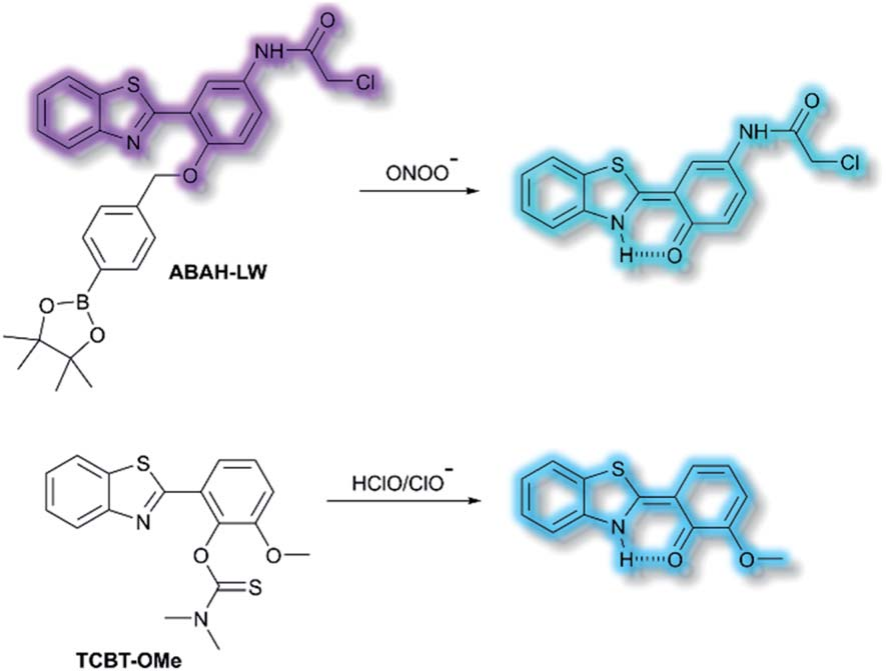
The structures of fluorescent probes **ABAH-LW** and **TCBT-OMe**.

James and co-workers have developed an ESIPT-based ratiometric fluorescent probe **ABAH-LW** that facilitates the detection of ONOO^−^ (Scheme 21).^88^**ABAH-LW** was able to detect ONOO^−^ within seconds and exhibits good selectivity and sensitivity towards ONOO^−^ over other ROS and biologically relevant analytes. Initially, the benzyl boronic ester ‘‘protecting’’ unit of **ABAH-LW** blocks the ESIPT process. On exposure of **ABAH-LW** to ONOO^−^, oxidative deprotection of the benzyl boronic ester unit occurs and leads to a ratiometric fluorescence response (*I*_481nm_/*I*_405nm_) with significant fluorescence enhancement (103-fold) in PBS buffer (8% DMSO, 1 mM CTAB, pH 8.2). Furthermore, the reactive chloroacetamide functional group of **ABAH-LW** enables covalent attachment to biomacromolecules located at the ER enabling the visualization of ONOO^−^ within the ER. **ABAH-LW** was shown to be cell permeable, which enabled it to be used to visualize ONOO^−^ in live HeLa cells. The same group also developed a HBT-based fluorescent probe **TCBT-OMe** for the detection of HOCl/ClO^−^ (Scheme 21).^89^ The presence of HClO/ClO^−^ leads to the hydrolysis of the dimethylthiocarbamate moiety resulting in a large increase in fluorescence intensity (∼42 fold) at *λ*_em_ = 472 nm (*λ*_ex_ = 310 nm). Probe **TCBT-OMe** was shown to have high sensitivity (LoD = 0.16 nM) and selectivity towards HClO/ClO^−^ over other ROS/RNS and can be used to detect endogenous and exogenous HClO/ClO^−^ in HeLa cells. In addition, test strips containing **TCBT-OMe** were constructed and could be used to detect HClO/ClO^−^ in drinking water. Furthermore, **TCBT-OMe** was used as dual input logic gate for Hg^2+^ and H_2_O_2_. Interestingly, the addition of Hg^2+^ alone to **TCBT-OMe** resulted in a significant increase in the fluorescence intensity (within 30 min), which is believed to due to the instability of the dimethylcarbamate (incorrectly written as dimethylcarbonate in the original paper) formed from the reaction of **TCBT-OMe** with Hg^2+^.

**Scheme 22:**
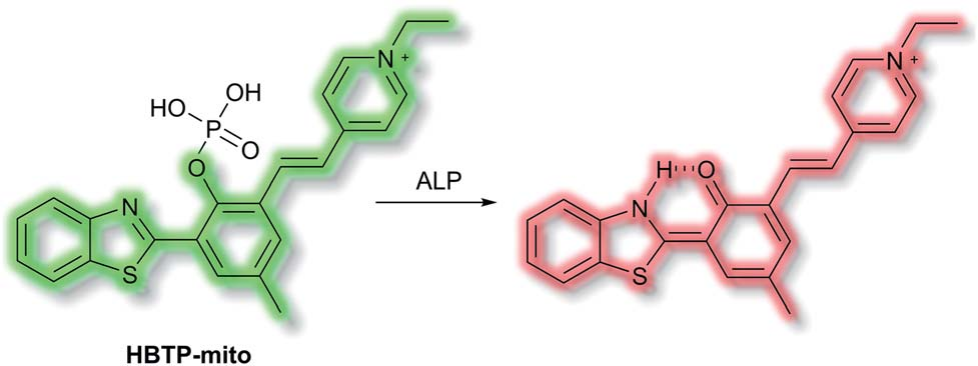
The structures of fluorescent probe **HBTP-mito**.

Zhang *et al.* have developed a novel mitochondria-targeted HBT-based ESIPT fluorescent probe **HBTP-mito** that facilitates the detection of ALP.^90^**HBTP-mito** is comprised of the HBT fluorophore and the pyridinium salt as a mitochondrial targeting group (Scheme 22). Initially, the ESIPT process of the phosphorylated probe is blocked and it exhibits fluorescence at *λ*_em_ = 514 nm. However, in the presence of ALP, hydrolysis of the phosphate ester and cleavage of the P–O bond releases HBT resulting in ESIPT turn on, and the emission switches from green (*λ*_em_ = 514 nm) to red (*λ*_em_ = 650 nm, Scheme 22) in TBS buffer solution (pH 8.0). Probe **HBTP-mito** exhibited high sensitivity (LoD = 0.072 mU mL^−1^) and excellent selectivity for ALP over other anions, and ions. In addition, the *I*_514nm_/*I*_650nm_ ratiometric response of the enzymatic hydrolysis product prevented fluorescence interference from the serum. Therefore, it could be used for detecting ALP in serum samples. Due to the low toxicity of **HBTP-mito**, it was suitable for monitoring ALP activity *in vitro* and *in vivo* and provided a blue-print for constructing ratiometric probes with longer wavelength emission and high sensitivity.

**Scheme 23:**
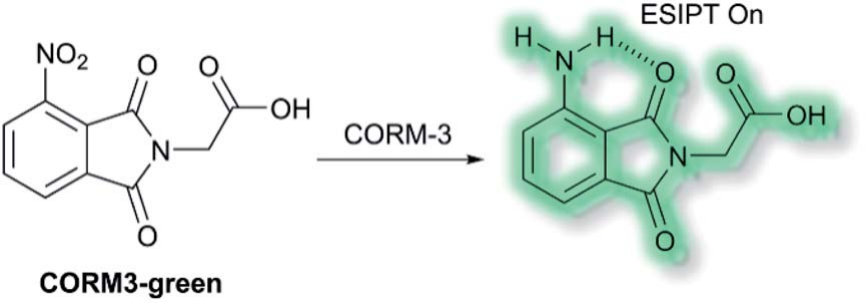
The structures of fluorescent probes **CORM3-green**.

Recently, Feng and co-workers have developed a fluorescent probe **CORM3-green** based on an ESIPT phthalimide dye for the detection of CORM-3 in both solution and living systems.^91^ CO-Releasing molecules (CORMs) are agents that release and deliver CO into the cell and can be used to replace the direct use of CO gas in the treatment of diseases.^92^**CORM3-green** is non-fluorescent, however, upon addition of CORM-3, the nitro group is reduced into an amino group to form a highly fluorescent phthalimide, which emits bright green fluorescence at *λ*_em_ = 503 nm in PBS buffer (0.5% DMSO, pH 7.4) solution due to the ESIPT process (Scheme 23). Furthermore, **CORM3-green** exhibited high sensitivity toward CORM-3 with a LoD of 16 nM, and excellent selectivity over other anions, metal ions, amino acids, ROS/RNS and biothiols including cysteine, glutathione and homocysteine. **CORM3-green** was evaluated in solution and in HeLa cells, zebrafish, and mice, thus demonstrating its use as a potentially powerful visualizing agent for biological applications.

**Scheme 24:**
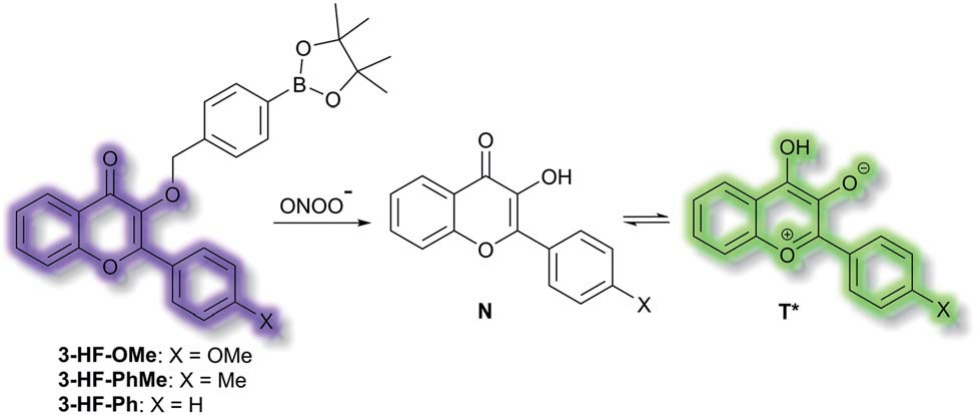
ESIPT probes **3-HF-X** (X = OMe, Me, H) for detecting ONOO^−^. The normal (N) and phototautomeric (T*) forms are shown.

**Fig. 3:**
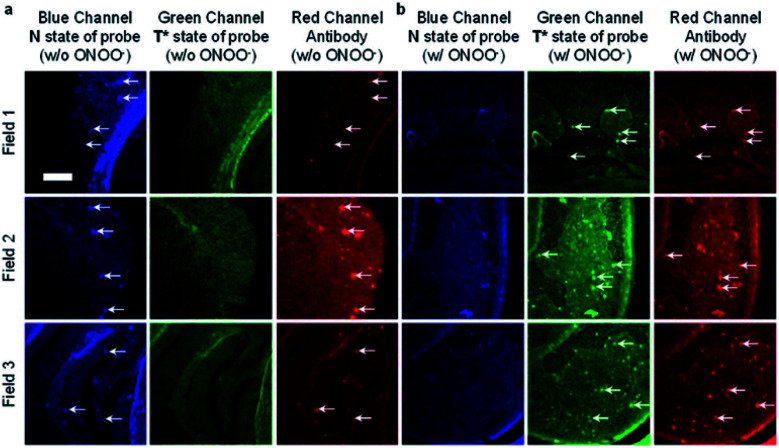
Fluorescence imaging of a brain section of a transgenic mouse treated with **3-HF-OMe** (20 μM) (a) without and (b) with ONOO^−^ (30 μM). The excitation/emission wavelengths for the blue (N-state), green (T*-state), and red (anti-Aβ antibody) channels are 404/425−475, 404/500−550, and 561/640−730 nm, respectively. The white arrows indicate stained Aβ aggregates. Reprinted from ref. 93. Copyright 2018, American Chemical Society.

James *et al.* have reported a series of 3-hydroxyflavone (3-HF) ESIPT boronate-based fluorescent probes that exhibit a ratiometric response toward ONOO^−^ in micellar environments (Scheme 24).^93^ Probe **3-HF-OMe**, is particularly sensitive toward hydrophobic environments and is able to differentiate between micellar and aqueous environments. The benzyl boronic ester ‘‘protecting’’ group blocks the ESIPT process, and the group can be selectively removed by ONOO^−^. Therefore, probe **3-HF-OMe** was found to produce a ratiometric fluorescence response when bound to Aβ aggregates (hydrophobic environment) in the presence of ONOO^−^. To investigate the potential of the probe for real world applications, fluorescence imaging of **3-HF-OMe** exhibited N-state fluorescence in mice brain slices (Fig. 3, blue channel) associated with Aβ aggregates as shown by correlation with anti-Aβ42 antibodies (Fig. 3, red channel). Then addition of ONOO^−^ to the brain sections, generated the T* state of **3-HF-OMe** (Fig. 3, green channel), which correlated well with the antibody fluorescence. As such these preliminary biological imaging experiments indicate how similar but longer wavelength ESIPT-based probes could be used to monitor fibrous proteins/peptides and environmental ROS/RNS. This system was described by the authors as a “reactive species” and “environment” based fluorescent probe.

Due to the unique transient nature of the four-level photochemical process of ESIPT, the ESIPT emission is highly sensitive to its local surroundings. Moreover, ESIPT fluorophores exhibit unusually large Stokes shifts (∼200 nm), when compared to traditional fluorophores (fluorescein, rhodamine, *etc.*). This helps avoid unwanted self-reabsorption and inner-filter effects. The limitation of ESIPT-based fluorescent probes come from the presence of polar and hydrogen bond donor solvents, which can lead to inhibition of the ESIPT process with no ketone (K*) emission.^13^ However, their use in combination with other fluorescence mechanisms will allow the construction of new probes that overcome the limitations of many current detection methods, while allowing the development of more and more practical fluorescence-based sensors and imaging agents.^93^

## 6. Aggregation induced emission (AIE) probes

Ordinarily, aggregation of fluorescent probe molecules is considered detrimental, resulting in aggregate-caused quenching (ACQ). The ACQ process is often exacerbated when a probe has multiple conjugated aromatic rings due to the increased formation of exciplexes and excimers.^94^ However, in 2001 the Tang group reported that 1-methyl-1,2,3,4,5-pentaphenylsilole exhibited the opposite effect – upon aggregate formation, a significant enhancement in photoluminescence was observed.^95^ The emission was ascribed to the severe steric hindrance of the fluorophore – since upon aggregation, the compound was unable to adopt a uniformly planar conformation. The process was termed ‘aggregation-induced emission’, and since then the area has expanded exponentially. Further investigations have elucidated that one common mechanism of AIE is the restriction of intramolecular motion (RIM).^96^ Such probes show negligible fluorescence in solution and exhibit a significant ‘turn-on’ upon aggregation. Common application of AIE compounds include use in electroluminescent devices and use as fluorescent sensors and dosimeters.^97^ In particular, AIE has been used in the field of biological fluorescence, for example in cell imaging, tumour imaging, and enzyme detection.^98^ Such probes are often referred to as ‘AIEgens’. The Tang group have published comprehensive reviews on, and extensive descriptions of, the AIE phenomenon, in particular their 2015 article ‘Aggregation-Induced Emission: Together We Shine, United We Soar’.^14^

**Scheme 25:**
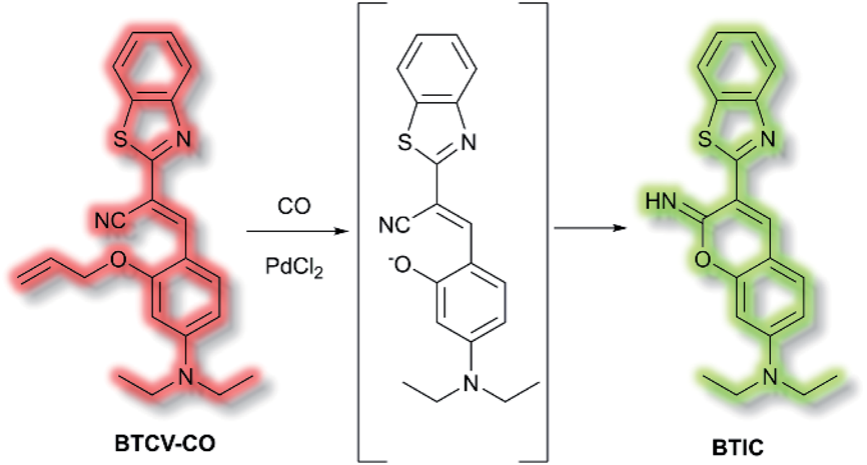
Detection of CO by AIEgen **BTCV–CO**.

In 2019, Tang *et al.* published an AIE probe for imaging carbon monoxide (CO).^99^ Whilst known for its toxicity, CO is also an endogenous gasotransmitter and is involved in a range of disease states and biological pathways. The probe, **BTCV–CO**, was designed to undergo a Tsuji–Trost reaction in the presence of CO and Pd^2+^ (Scheme 25). In solution, **BTCV–CO** was found to fluoresce at *λ*_em_ ≈ 675 nm. Solution aggregation experiments with the probe confirmed the AIE characteristics. Mechanistically, it has been proposed that in solution rapid non-radiative decay can occur from the singlet excited state, mediated by *cis–trans* isomerisation of the trisubstituted alkene. Then, upon aggregation the isomerisation pathway is hindered. Therefore, formation of **BTIC***via* the Tsuji–Trost reaction would similarly prevent this *cis*–*trans* isomerisation. Studies in PBS (5% DMSO, v/v, pH 7.4) with CO donor [Ru(CO)_3_Cl_2_]_2_ (CORM-2) and PdCl_2_ confirmed the expected fluorescent enhancement. On formation of **BTIC**, a new emission band at *λ*_em_ = 546 nm was observed, facilitating the ratiometric (*I*_546nm_/*I*_710nm_) detection of CO. The ratiometric response of **BTCV–CO** to CO was more sensitive than just the turn-on response, affording fluorescent enhancements of 39-fold and 17-fold respectively. The ratiometric enhancement was found to correlate with increasing concentrations of CORM-2, which scaled linearly at low concentrations (1–5 μM). Furthermore, the probe was found to be selective towards CO over a range of biological analytes (including ROS, RNS, amino acids, and common anions), and the detection limit of **BTCV–CO** towards CO was determined to be 30.8 nM. Exogenous addition of CO (*via* CORM-2) to cells loaded with **BTCV–CO** confirmed that the cells underwent fluorescent enhancement in the green window (*λ*_ex_ = 476 nm, *λ*_em_ = 500–580 nm) relative to cells loaded with only the probe which showed weak emission in both the green and red (*λ*_em_ = 580–700 nm) windows. Enhancement of the ratiometric response was found to correlate with the concentration of CO added. Furthermore, *in vivo* evaluation confirmed that a significant fluorescent response could be achieved in mice injected with **BTCV–CO** + CORM-2 + PdCl_2_, whereas injection with only **BTCV–CO** + PdCl_2_ resulted in only weak emission.

**Fig. 4:**
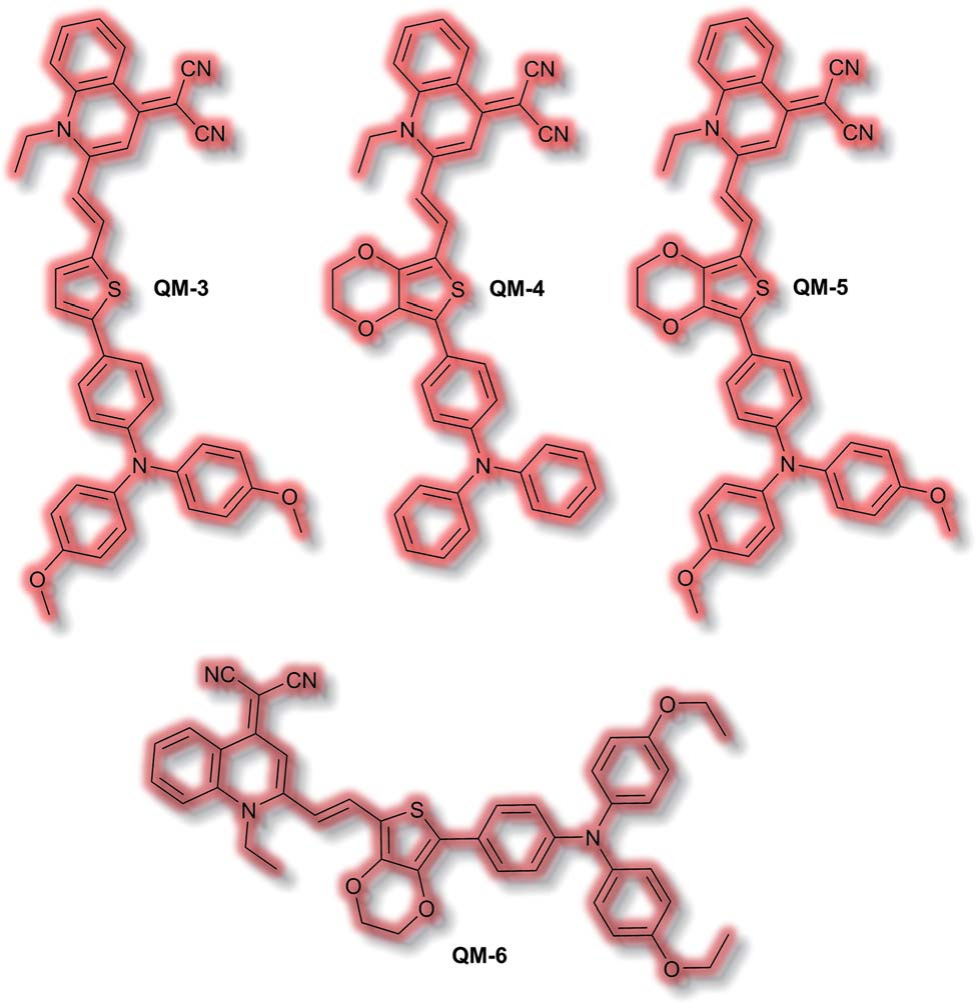
Structures of AIEgens **QM-3**–**QM-6**.

An extensive area of the NIR AIE literature concerns probes for tumour sensing and imaging, particularly as contrast agents.^100,101^ In 2015, Zhu *et al.* designed a series of tumour-targeting probes based on quinoline–malononitrile AIE fluorophores conjugated to a range of electron-donating motifs *via* thiophene linkers (Fig. 4).^102^ Probes **QM-3** to **QM-6** were found to exhibit NIR AIE behaviour in solution. Notably, increasing the electron-donating character of the triphenylamine moiety in **QM-3**, **QM-5**, and **QM-6** resulted in a redshift of the AIE. A 3,4-ethylene-dioxythiophene (EDOT) moiety was introduced to compounds **QM-4**, **QM-5**, and **QM-6** to tailor the AIE by altering the donor–π–acceptor system through the resultant change in conformation. This resulted in increased aggregation control with the solution-based studies. Imaging of evaporation experiments, to simulate aggregation, revealed that **QM-3** adopted a microrod structure whilst **QM-4–QM-6** formed spherical nanoparticles. The difference in morphology was attributed to the introduction of the EDOT moiety in the latter species. Furthermore, the photostability of each species was found to be ≈20-fold greater than commercially available dye ICG. **QM-5** exhibited the most desirable characteristics and was evaluated in cells. The probe underwent rapid uptake and spherical aggregate formation in HeLa cells. Furthermore, **QM-5** remained fluorescent 24 h after incubation and was emissive for twice as many cell passages as the ICG control. *In vivo* experiments in mice indicated that **QM-5** was tumour specific, affording an AIE response within 30 minutes. Furthermore, the probe was tumour retentive, remaining in tumour tissue for 24 h. The tumour specificity was ascribed to the enhanced permeability and retention (EPR) effect afforded by the spherical shaped aggregates. *Ex vivo* organ imaging of injected mice indicated that whilst **QM-5** aggregated in tumour tissue, it also aggregated in the liver.

**Scheme 26:**
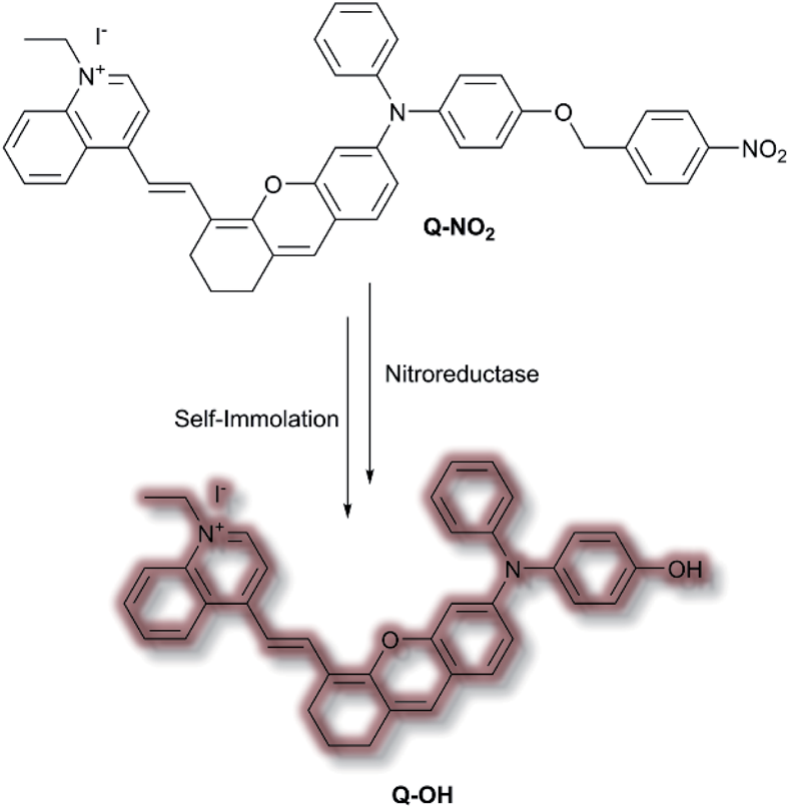
Nitroreductase detection mechanism by AIEgen **Q-NO2**.

In 2020, Wu *et al.* designed an NIR probe for the detection of breast cancer metastasis, combining AIE and multispectral optoacoustic tomography (MSOT).^103^ The group used a donor–π–acceptor system of dihydroxanthene and quinolinium groups respectively, using a nitroaryl recognition moiety to afford **Q-NO2**. Upon reduction of the nitro group by nitroreductase, the resultant amine undergoes self-immolation of the *p*-benzyl moiety to afford phenol **Q-OH** (Scheme 26). Fluorescent enhancement occurred at *λ*_em_ = 780 and 922 nm when excited at *λ*_ex_ = 680 and 808 nm respectively due to the formation of **Q-OH** aggregates; the fluorescence intensity at each emission band was found to increase linearly with increasing concentrations of nitroreductase. Furthermore, a linear optoacoustic response with increasing nitroreductase was observed. The probe was found to be highly selective over a range of relevant biological analytes, and a detection limit of 0.052 μg mL^−1^ towards nitroreductase was established. For cell testing, 4T1 murine mammary carcinoma cells were chosen due to their overexpression of nitroreductase. Hypoxic cells, known to overexpress nitroreductase, stained with **Q-NO2** displayed a turn on-response for both the NIR-I and NIR-II window, compared to control cells which exhibited minimal fluorescence. In addition, an optoacoustic response that mirrored that of the fluorescence response was observed. **Q-NO2** was shown to detect orthotopic and far metastatic tumours in the mouse models used, with an enhanced NIR-I and NIR-II response relative to the bioluminescence control. Tumour imaging *via* the NIR-II window afforded a clearer image of the tumours due to the decreased autofluorescence. Notably, MSOT imaging of mice with **Q-NO2** was able to effectively monitor both orthotopic tumour growth and eventual metastasis in 3D.

**Fig. 5:**
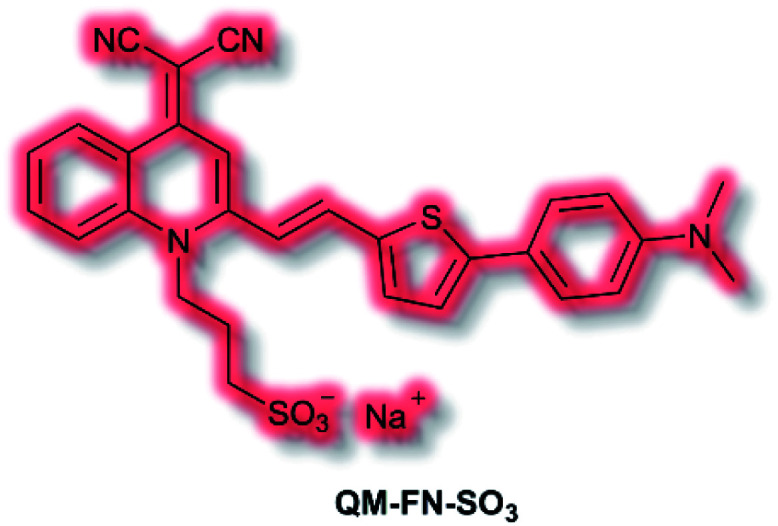
Structure of AIEgen **QM-FN-SO3**.

Aβ is another key biological target, due to its role in the pathogenesis of Alzheimer's disease. In 2019, Zhu *et al.* described a new NIR AIE probe for the imaging of Aβ plaques.^104^ The authors sought to design a probe that improved on commercial probe Thioflavin T (ThT), which suffers from ACQ, low signal-to-noise ratio, and low BBB penetration. Probe **QM-FN-SO3** incorporated the quinoline–malononitrile NIR fluorophore, to which a conjugated thiophene was attached to increase lipophilicity and aid BBB penetration. In addition, a sulfonate group was added to increase water solubility and generate a ‘fluorescence-off’ state when not aggregated (Fig. 5). Probe **QM-FN-SO3** was non-fluorescent in water but a turn-on fluorescence response (*λ*_ex_ = 500 nm, *λ*_em_ = 720 nm) was observed as the solubility of the probe decreased and aggregation began. In PBS solution, **QM-FN-SO3** displayed a positive AIE response to Aβ aggregates, for which fluorescent enhancement (blue-shifted to *λ*_em_ = 665 nm) increased as the concentration of Aβ aggregates increased. Mechanistically, it was proposed that the *N*-dimethylamino group acts as a recognition moiety, which once bound experiences reduced freedom of rotation, affording a fluorescence response. The half-life of **QM-FN-SO3** was found to be ≈5× that of ThT at ≈40 min and exhibited a greater signal-to-noise ratio both in solution and in mouse brain models evaluated. Furthermore, *in vivo* evaluation using mouse models indicated that could **QM-FN-SO3** cross the BBB and produce a fluorescence response to Aβ plaques in the hippocampus.

An obvious advantage for the AIE strategy is the ability to overcome the quenching issues presented by ACQ. Furthermore, for turn-on AIEgens, the probe is non-fluorescent in solution. As a fluorescent signal is only observed upon aggregation, as such there is no overlap between the signal of the species involved in the sensing process, and the process is independent of the probe concentration.^94^ Due to the size of their extensive π-systems, some AIEgens can suffer from increased hydrophobicity, which may lead to issues with cellular uptake and distribution.^105^

## 7. Multiple modality fluorescent probes

Fluorescent chemosensors have been widely applied in diverse fields such as biology, physiology, medicine, and pharmacology. Fluorescence output signals can be observed using optical instruments and even using the naked eye in real-time.^16^ Fluorescent chemosensors generally exhibit changes in fluorescence intensities and wavelength using various single sensing mechanisms, including ICT, PeT, FRET, ESIPT and AIE.^12,50,106,107^ However, fluorescent chemosensors can also be designed based on dual (or multiple) fluorescence mechanisms in order to deliver diverse fluorescence outputs, facilitating the simultaneous tracking of multiple analytes or improving the selectivity and sensitivity for a particular analyte.

**Scheme 27:**
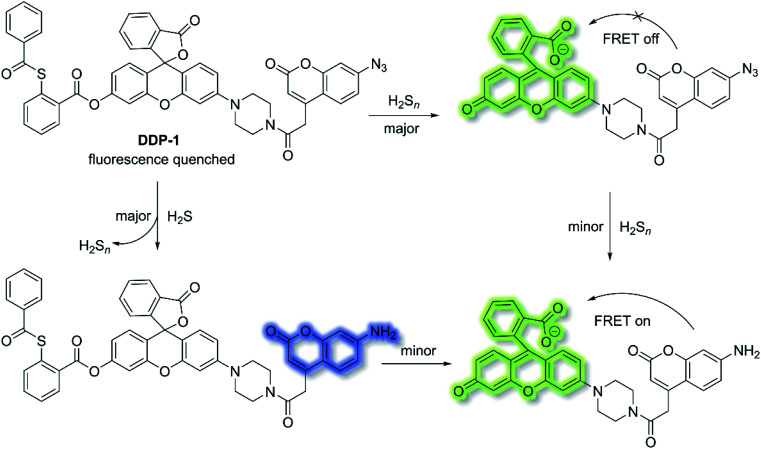
**DDP-1** detection of H_2_S and H_2_S_*n*_ using two distinct emission channels.

H_2_S and hydrogen polysulfides (H_2_S_*n*_, *n* > 1) are endogenous regulators of many physiological processes,^108^ and data suggest that H_2_S_*n*_ might be the main signalling molecules instead of H_2_S. In order to reveal the mutual relationship and cellular cross-talk between H_2_S and H_2_S_*n*_ in cells, Xian *et al.* reported a dual-channel fluorescent probe **DDP-1** using a rhodol-coumarin hybrid for visualizing H_2_S and H_2_S_*n*_ using different fluorescence signals.^109^ With **DDP-1**, an azidocoumarin moiety and phenyl 2-(benzoylthio)benzoate were chosen as the reaction sites for H_2_S and H_2_S_*n*_. When H_2_S_*n*_ reacts with the phenyl-2-(benzoylthio)benzoate, the green fluorescence of rhodol at *λ*_em_ = 542 nm (*λ*_ex_ = 360 nm) was observed (Scheme 27). However, on the addition of H_2_S, the situation is more complicated since the reaction between H_2_S and azides led to the formation of H_2_S_*n*_, however, less than 0.5 equivalents of H_2_S_*n*_ are produced from the reaction of H_2_S (1 equivalent) and azide, which means the reaction with H_2_S produced both the blue coumarin emission at *λ*_em_ = 445 nm (major) and green rhodol emission at *λ*_em_ = 542 nm (minor) (*λ*_ex_ = 360 nm). Therefore, **DDP-1** can detect H_2_S and H_2_S_*n*_ using these two distinct emission channels. As such **DDP-1** could be used for the detection of H_2_S and H_2_S_*n*_ in HeLa cells.

**Scheme 28:**
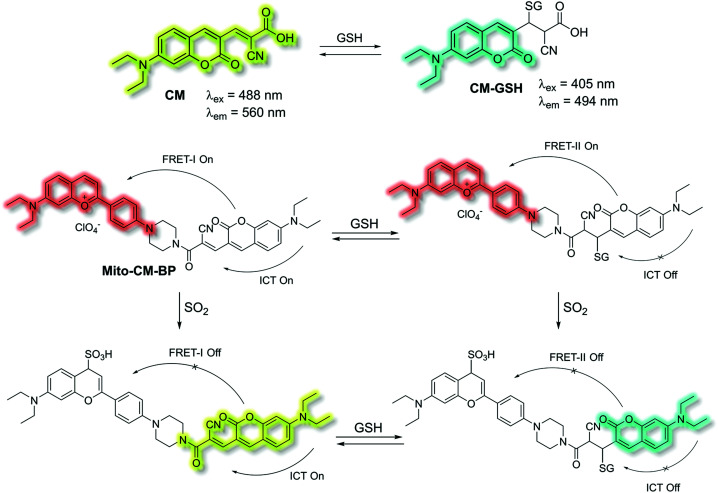
ICT and FRET-based probe **Mito-CM-BP** developed to allow for the simultaneous detection of GSH and SO_2_.

Yin *et al.* have developed a novel dual-functional fluorescent probe **Mito-CM-BP** based on an ICT-FRET mechanism for the simultaneous detection of GSH and SO_2_ (Scheme 28).^110^ The probe consists of an ICT based coumarin–cyanoacetic acid system as an energy donor and a reaction site for GSH; while the benzopyrylium unit (BP) can not only serve as an energy acceptor but also as a sensitive reaction site for SO_2_. Two excitation wavelengths at *λ*_ex_ = 488 and *λ*_ex_ = 405 nm, which correspond to **CM** (donor of FRET-I) and **CM-GSH** (donor of FRET-II), were chosen as there are two different FRET systems before and after **Mito-CM-BP** reacts with GSH. The free probe **Mito-CM-BP** displayed a fluorescence emission at *λ*_em_ = 638 nm (*λ*_ex_ = 488 nm). Then upon addition of SO_2_, the FRET-I process of **Mito-CM-BP** was blocked, and the fluorescence emission at 638 nm gradually decreased and a shorter wavelength emission at 560 nm appeared. Conversely, exposure of **Mito-CM-BP** to GSH resulted in destruction of the π-conjugation between coumarin and cyanoacetic acid. The inhibition of the ICT process triggers a FRET-II process from the **CM-GSH** donor to the BP acceptor, leading to a significant fluorescence enhancement at *λ*_ex_ = 638 nm (*λ*_ex_ = 405 nm). Furthermore, addition of SO_2_ breaks the conjugated system of the BP moiety, which stops the FRET-II process, resulting in an increase in emission at *λ*_em_ = 494 nm and decrease in emission at *λ*_em_ = 638 nm. Importantly, **Mito-CM-BP** has been successfully used for the visualization of enzymatic conversion of intracellular GSH to SO_2_ in different cell lines (HepG2 and SW480 cells) and tumour-bearing mice, which may help to clarify the metabolic pathways of SO_2_ production and facilitate an understanding of the role of SO_2_ in biological systems.

**Scheme 29:**
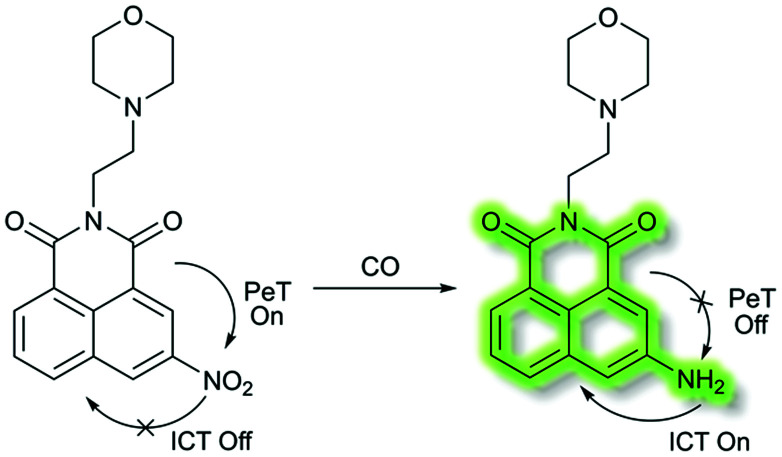
A lysosome targetable fluorescent probe **LysoFP-NO2** for detection of CO.

In 2018, Dhara *et al.* developed a lysosome targeting CO fluorescent probe **LysoFP-NO2**, consisting of a naphthalimide fluorophore, a nitro group as the CO responsive moiety, and a morpholine fragment as the lysosome-targeting unit (Scheme 29).^111^**LysoFP-NO2** displayed weak fluorescence due to the strong electron withdrawing ability of the nitro group, which can quench the fluorescence of the fluorophores by PeT. However, upon the addition of CO, the nitro group was converted into an amino moiety, stopping PeT and strengthening the ICT process. Therefore, probe **LysoFP-NO2** displayed a “turn on” fluorescence response towards CO with a 75-fold fluorescence enhancement in HEPES buffer (1% DMSO, pH 7.4). In addition, probe **LysoFP-NO2** displayed high selectivity over other relevant reactive nitrogen, oxygen, and sulfur species. Moreover, **LysoFP-NO2** could be used to monitor changes in intracellular CO within the lysosomes of live MCF7 cells.

**Scheme 30:**
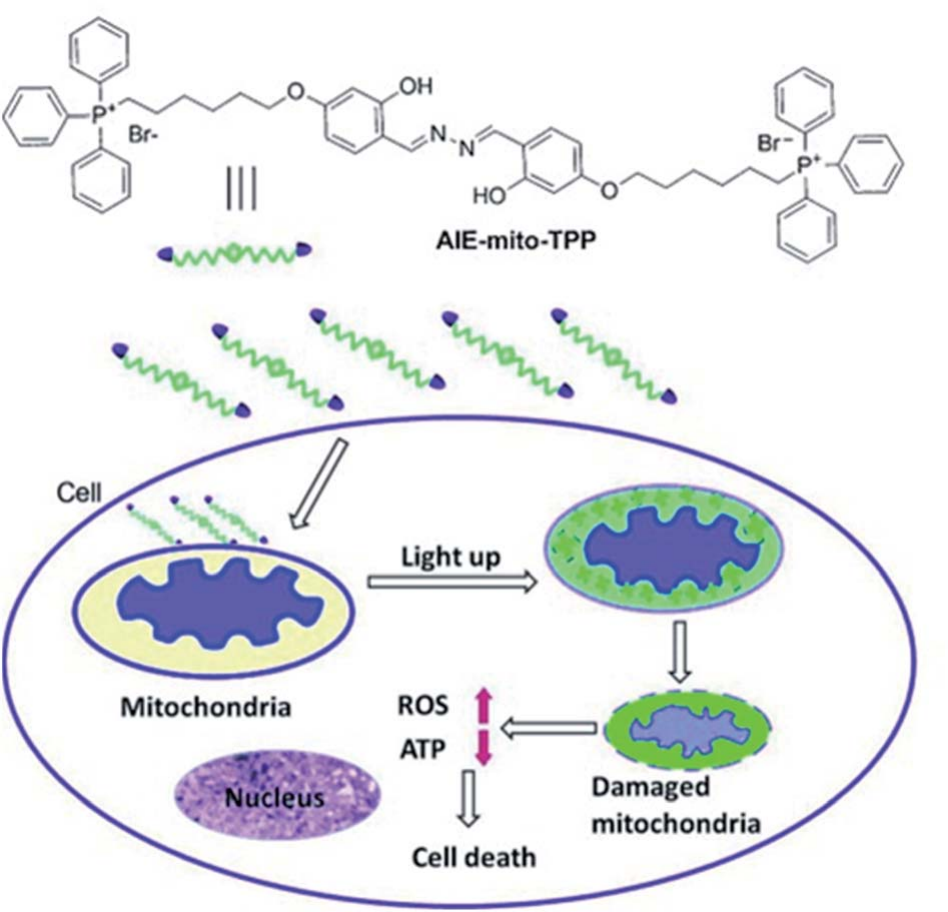
The structure of probe **AIE-mito-TPP** and schematic representation of intracellular tracking and the therapeutic effect of **AIE-mito-TPP** in cancer cells. Reprinted from ref. 112. Copyright 2014, Wiley-VCH Verlag GmbH & Co. KGaA, Weinheim.

Liu *et al.* have developed a mitochondria-targeting probe **AIE-mito-TPP** based on an AIE and ESIPT system as an imaging agent and potential chemotherapeutic drug.^112^ Triphenylphosphonium (TPP), a positively charged lipophilic cation, is a well-known mitochondrial targeting ligand due to the negative potential gradient of the organelle.^113^**AIE-mito-TPP** consists of a salicylaldazine fluorophore attached to two TPP groups for targeting the mitochondria (Scheme 30). The salicylaldazine fluorophore uses two emission mechanisms: AIE *via* restriction of intramolecular rotation around the N–N bond and ESIPT caused by intramolecular hydrogen bonds. **AIE-mito-TPP** was almost non-fluorescent in cell-culture media but the fluorescence due to both AIE and ESIPT is activated in mitochondria (*λ*_ex_ = 543 nm, *λ*_em_ = 575–625 nm). Furthermore, **AIE-mito-TPP** accumulated preferably in cancer cell mitochondria and exhibited selective cytotoxicity towards cancer cells which was attributed to the difference in mitochondrial membrane potential between normal and cancerous cells. In addition, **AIE-mito-TPP** could induce mitochondrial dysfunction influencing several important cellular processes and facilitating the selective killing of HeLa cells.

**Scheme 31:**
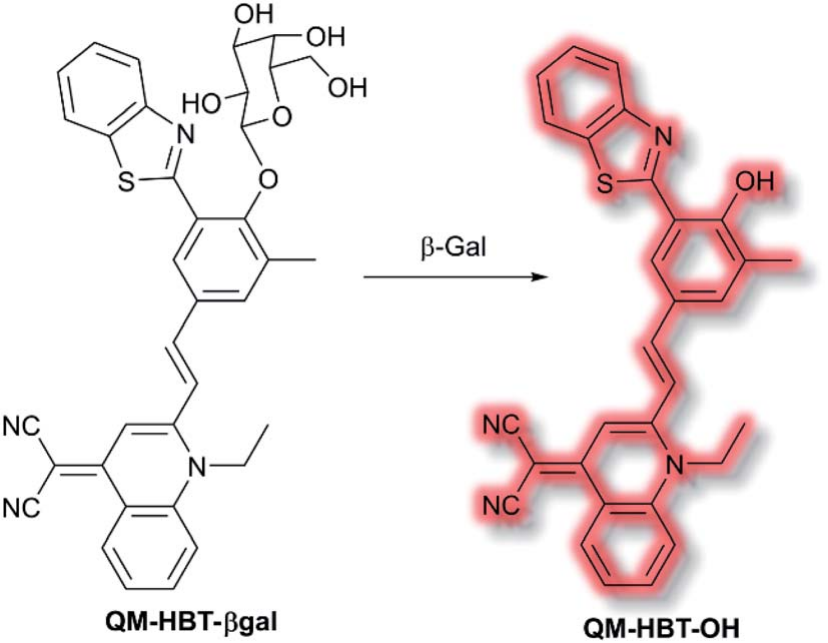
Detection of *β-*gal by combined AIE-ESIPT probe **QM-HBT-βgal**.

As discussed in the section on ICT probes, *β*-gal is a known biomarker for ovarian cancer. In 2019, Guo *et al.* reported a probe for monitoring *β*-gal activity, designed around the commonly used AIE quinoline–malononitrile fluorophore.^114^ A 2-(2-hydroxyphenyl)benzothiazole moiety was conjugated to both increase lipophilicity and extend the π-system to favour NIR emission. A galactose residue was attached to the probe to act as the recognition moiety, to give the probe **QM-HBT-βgal**. In PBS solution (30% DMSO, v/v, pH 7.4) **QM-HBT-βgal** produced a turn on response at *λ*_em_ = 650 nm (*λ*_ex_ = 460 nm) in the presence of *β*-gal. It was proposed that the saccharide moiety of **QM-HBT-βgal** increased the hydrophilicity of the probe; and upon cleavage by *β*-gal, the resulting **QM-HBT-OH** was insoluble in solution and aggregated to give an AIE response (Scheme 31). Furthermore, cleavage of the sugar moiety unmasks the ESIPT system upon formation of the hydroxyl moiety. An increase in emission at 650 nm occurred, scaling linearly, with added *β*-gal. Furthermore, the probe was found to be highly selective (over other assayed enzymes and amino acids), was stable over a pH range from 3 to 11, and exhibited a 60-fold greater half-life than commercial standard ICG. Due it is excellent performance in solution, **QM-HBT-βgal** was then evaluated for the *in vivo* detection of *β*-gal. **QM-HBT-βgal** exhibited no fluorescence in HeLa cells without exogenous addition of *β*-gal, whereas SKOV-3 cells exhibited significant fluorescence enhancement with the probe due to high endogenous *β*-gal expression. Inhibition studies confirmed that the AIE response originated from *β*-gal activity.

**Scheme 32:**
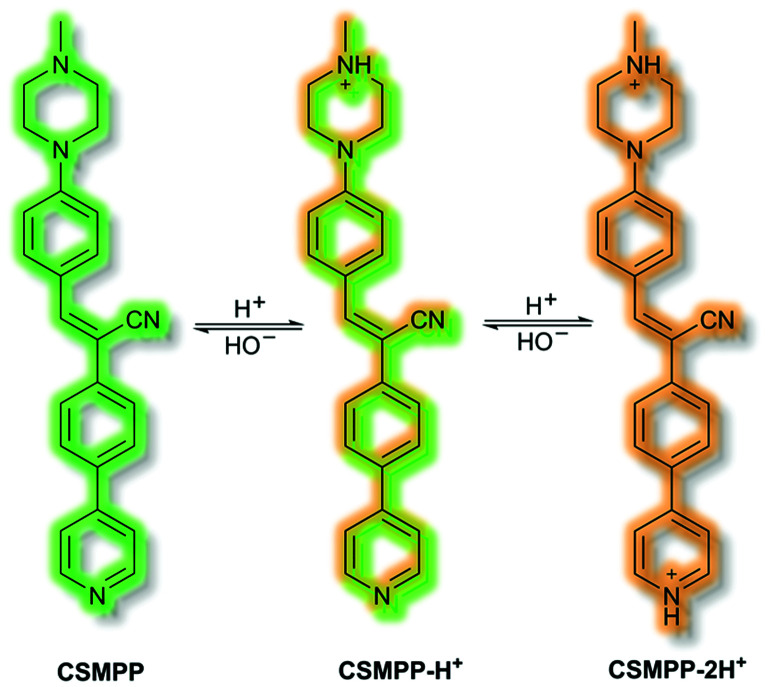
Combined AIE-ICT probe **CSMPP**.

Recently, in 2020 the Tang group proposed an AIE lysosome targeting probe for monitoring lysosomal pH during the tissue regeneration process.^115^ Lysosomes play a key role in tissue regeneration, for which pH 4–5 is essential for lysosomal hydrolase activity. Whilst research has been conducted on lysosomes and tissue regeneration, an *in vivo* study of lysosome pH during the regeneration process had not previously been reported. Probe **CSMPP** was designed around an α-cyanostilbene fluorophore, to which a piperazine moiety was attached to target the lysosome. In solution (MeCN), **CSMPP** was found to be weakly fluorescent at *λ*_em_ = 509 nm (*λ*_ex_ = 365 nm) but the fluorescence enhanced upon addition of water (MeCN/water, 2/8, v/v) due to aggregation. By varying the pH of **CSMPP** in solution, a ratiometric emission response was observed; relative to neutral solutions, at acidic pH the emission band at *λ*_em_ = 509 nm decreased and a new emission at *λ*_em_ = 615 nm increased. The process was found to be reversible. The acid sensitivity of the probe was ascribed to the piperazine and pyridine moieties (Scheme 32). Furthermore, DFT calculations indicated that protonation of the pyridine moiety afforded enhanced ICT, imparting a ratiometric response upon protonation. Staining of HeLa and ARPE-19 cells confirmed that **CSMPP** could selectively target lysosomes, with Pearson coefficients of 0.92 and 0.90 respectively. *In vivo* NIR confocal studies with medaka larvae indicated that **CSMPP** was able to target lysosomes in the larval fin with good specificity. **CSMPP** was then used to study the regeneration process of injured medaka caudal fins *in vivo*. Relative to a control group which saw almost no lysosomal pH change, healing caudal fins were shown to undergo a range of pH changes post-amputation. Within 24 h, the pH dropped from 5.1 to 4.6 due to increased lysosomal activity. Interestingly, the pH of lysosomes adjacent to the injury were found to be more acidic than those further away. As the tissue regenerated, the pH returned to normal after 5 days.

Compared to single sensing mechanism-based sensors, dual/triple sensing mechanism-based sensors generally produce changes in the emission intensity and wavelength of the signals at the same time, which can provide different fluorescence signals or amplify the response signals.^16^ This is highly desirable for the simultaneous monitoring of multiple analytes or improving the selectivity and sensitivity of fluorescence sensors. Furthermore, the signal-to-noise ratio would significantly increase when the two/multiple mechanisms have similar effects on the same chromophore. We anticipate that dual and multi mechanism based fluorescent sensors will be employed for exciting applications in various fields, especially in high signal-to-noise ratio fluorescence bioimaging, investigating interactions among multiple biological species, and the development of optical logic devices with multiple inputs and outputs.

## 8. Conclusions

Over the last decade there have been considerable advances in the development of molecular fluorescent probes used for biological sensing and imaging, advancing our understanding and guiding our treatment of a diverse range of medical conditions. In particular, the development of high-resolution techniques including STORM, PALM and PAI *etc.* have invigorated the area by encouraging the *de novo* design of probes with improved photophysical properties and targeting ability. With this perspective we have highlighted some of the recent developments made in the design of such fluorescent probes, using the following approaches: FRET; ICT; PeT; ESIPT; AIE and probes combining these modalities. Each probe has been selected based on its ability (or potential), for detecting analytes of interest in a real-world environment. In addition, we have highlighted the advantages and disadvantages of each fluorescence based approach. We end with a section outlining how some disadvantages of individual fluorescence based systems can be overcome through the combination of multiple fluorescence techniques.

In summary, with this perspective we believe we have highlighted recent progress made in the development of fluorescent probes using singular and multiple modality fluorescence approaches. From this perspective, it is clear that the area of fluorescent probes used for biological applications will continue to be challenged and pushed forward by the development of enhanced measurement techniques, that require improved photophysical properties and targeting ability, beyond what is currently available.

## Conflicts of interest

There are no conflicts to declare.
